# Immune remodeling and metabolic reprogramming in chronic fatigue: insights into GPCR signaling and epigenetic regulation

**DOI:** 10.3389/fimmu.2026.1806420

**Published:** 2026-05-15

**Authors:** Zekai Hu, Jinyan Wang, Sicong Ma, Jie Zhuang, Jing Shi, Yan Zhu

**Affiliations:** 1 Department of Rehabilitation Medicine, The Second Rehabilitation Hospital of Shanghai, Shanghai, China; 2 School of Medicine, Shanghai University, Shanghai, China; 3 Department of Rehabilitation Medicine, Renji Hospital Affiliated to Shanghai Jiao Tong University School of Medicine, Shanghai, China

**Keywords:** bioactive metabolites, chronic inflammation, epigenetic acylation, fatigue, GPCR signaling, histone modification, immunometabolism, mitochondrial dysfunction

## Abstract

Inflammation-driven fatigue is a clinically significant feature of several chronic inflammatory conditions, including myalgic encephalomyelitis/chronic fatigue syndrome (ME/CFS), post-COVID condition, autoimmune disease, and cancer-related fatigue. Across these conditions, partially overlapping disturbances in immune regulation, cellular metabolism, and neuroimmune signaling may contribute to persistent fatigue, despite important differences in initiating context and biological substrate. Current evidence implicates mitochondrial dysfunction, altered glycolysis and fatty acid utilization, lactate- and succinate-associated signaling, metabolite-sensing G protein-coupled receptor (GPCR) pathways, epigenetic acylation, and immune remodeling in the maintenance of fatigue. This narrative review synthesizes both shared and disease-context-specific mechanisms underlying inflammation-associated fatigue, with particular emphasis on immunometabolism, peripheral-central neuroimmune crosstalk, metabolite-GPCR signaling, and epigenetic regulation. We highlight GPCR signaling as a potentially important regulatory interface in inflammatory and metabolic pathways relevant to fatigue, while recognizing that direct causal evidence in human fatigue syndromes remains limited. The review also examines how metabolite-mediated epigenetic acylation may influence immune cell function and fatigue-related biology, although this association remains incompletely validated in fatigue-specific settings. By integrating metabolic dysregulation, neuroimmune signaling, and immune dysfunction, this review consolidates current knowledge on candidate biomarkers, mechanistic pathways, and emerging therapeutic targets in chronic inflammation-driven fatigue. Overall, this review provides a multidimensional framework for understanding fatigue across inflammatory disorders and for guiding future mechanistic and translational research.

## 1 Introduction

Chronic fatigue is a pervasive and debilitating symptom encountered across a spectrum of chronic diseases, significantly impairing patients’ quality of life and complicating disease prognosis. Traditionally, the pathophysiology of chronic fatigue has been predominantly attributed to inflammatory cytokines and dysregulation of neuroendocrine axes, such as the hypothalamic-pituitary-adrenal (HPA) axis. However, emerging evidence increasingly implicates alterations in cellular metabolism and bioactive metabolites as pivotal contributors to the inflammation-fatigue axis. For instance, in neurodegenerative disorders like Parkinson’s disease (PD), chronic fatigue syndrome (CFS) is prevalent in approximately two-thirds of patients and correlates strongly with elevated serum inflammatory markers including CCL5, sVCAM-1, and NCAM, underscoring the inflammatory underpinnings of fatigue in these conditions (1). Similarly, in the context of long COVID, a subset of patients presenting with myalgic encephalomyelitis/chronic fatigue syndrome (ME/CFS) exhibit distinct immunological dysregulation characterized by elevated pro-inflammatory cytokines, chemokines such as Galectin-9, and immunosuppressive erythroid cells, which collectively contribute to persistent fatigue and cognitive impairment (2). These findings highlight the intricate interplay between systemic inflammation and fatigue manifestations, suggesting that chronic inflammation is not merely a bystander but an active driver of fatigue pathogenesis.

Beyond inflammatory mediators, attention has turned to the role of metabolic pathways and their regulatory mechanisms in chronic fatigue. Multi-omics analyses in ME/CFS patients reveal impaired energy metabolism involving key biochemical circuits such as the citric acid cycle, beta-oxidation of fatty acids, and amino acid-derived urea cycle energy production. These metabolic derangements are compounded by systemic inflammation linked to lipid abnormalities, disrupted extracellular matrix homeostasis, and redox imbalances, which collectively exacerbate fatigue and post-exertional malaise (3). Moreover, alterations in tryptophan catabolism through the kynurenine pathway have been implicated in long COVID-related fatigue, where increased kynurenine-to-tryptophan ratios and insulin resistance correlate with severity of physiosomatic and affective symptoms (4). These metabolic perturbations are not isolated phenomena but are integrated with immune dysregulation and neuroendocrine dysfunction, reinforcing the concept of a multifactorial etiology for chronic inflammation-driven fatigue.

A critical mechanistic axis that has garnered increasing attention involves the interplay among metabolite-responsive G protein-coupled receptor (GPCR) signaling (5), epigenetic regulation including histone acylation (6), and immune-cell metabolic remodeling (7). GPCRs function as major transducers of extracellular cues, including signals derived from bioactive metabolites, and thereby participate in the regulation of inflammatory and metabolic responses (8).

GPCRs are integral to transducing extracellular signals, including those from bioactive metabolites, thereby modulating inflammatory and metabolic responses. Acetylation-dependent epigenetic regulation can influence gene-expression programs relevant to immune activation and metabolic adaptation, providing a dynamic interface between environmental signals and cellular function (9). During chronic inflammation, immune cells undergo metabolic reprogramming, often with a relative shift away from oxidative phosphorylation toward glycolysis or other metabolic states capable of sustaining inflammatory phenotypes (7). Together, these emerging insights provide a broader mechanistic framework for understanding how chronic inflammation may contribute to fatigue through coordinated changes in cellular metabolism and signaling networks (10). At present, however, much of the evidence linking metabolite-sensing GPCR pathways to fatigue remains indirect and is derived predominantly from mechanistic studies in cellular systems or animal models, rather than from direct causal demonstration in human fatigue phenotypes.

In this review, “inflammation-driven fatigue” is used as a mechanistic construct to describe fatigue arising in association with sustained inflammatory signaling, immune remodeling, and metabolic disturbance (11). This framework does not assume nosological equivalence among ME/CFS, long COVID, cancer-related fatigue, and autoimmune-associated fatigue. Rather, these conditions are considered together because partially overlapping alterations have been reported in cytokine signaling, mitochondrial homeostasis, metabolite sensing, and immunometabolic adaptation, despite major differences in initiating triggers and tissue context (12). Accordingly, the present review emphasizes convergent mechanisms without disregarding disease-specific biological heterogeneity. The relationship between ME/CFS and long COVID is characterized by both mechanistic overlap and disease-specific distinctions, as summarized in **Table 1**.

**Table 1:** Major overlapping and distinguishing features between ME/CFS and long COVID.

Domain	ME/CFS	Long COVID	References
Typical initiating context	Often follows an infectious illness or other physiological stressor; post-infectious onset is common but not universal	Develops after documented or probable SARS-CoV-2 infection	(2, 13, 14)
Core symptom overlap	Persistent fatigue, post-exertional malaise, cognitive dysfunction, sleep disturbance, and orthostatic intolerance are common	Persistent fatigue, exertional symptom exacerbation, cognitive dysfunction, dysautonomia, and sleep disturbance are also common	(2, 11)
Immune/inflammatory features	Chronic low-grade immune dysregulation, altered cytokine/chemokine signaling, and neuroimmune disturbance have been reported	Persistent immune activation and post-viral inflammatory remodeling have been reported in subsets of patients	(13–15)
Metabolic features	Disturbed energy metabolism, mitochondrial dysfunction, redox imbalance, and heterogeneous metabolomic abnormalities have been reported	Persistent metabolomic abnormalities and altered tryptophan-kynurenine metabolism have been reported	(3, 4, 13, 14)
Disease relationship	Distinct clinical entity with marked biological heterogeneity	Heterogeneous post-COVID syndrome; a subgroup overlaps with an ME/CFS-like phenotype, but the two conditions are not interchangeable	(11, 15)
Biomarker status	No single validated diagnostic biomarker is currently accepted for routine clinical use	No single validated diagnostic biomarker is currently accepted for routine clinical use	(128, 167, 168)

In the context of ME/CFS, chronic inflammation is unlikely to arise from a single uniform cause, but rather from a range of persistent or incompletely resolved inflammatory contexts. Among these, post-infectious immune dysregulation is considered a plausible initiating context, as some individuals may fail to return to immune and metabolic homeostasis after acute viral infection. Viral infections, including Epstein–Barr virus, enteroviruses, and SARS-CoV-2, have been associated with prolonged fatigue syndromes or ME/CFS-like phenotypes, supporting a role for post-viral processes in disease onset in susceptible individuals (13, 14). In this setting, viral infection is more appropriately regarded as a potential trigger rather than a universally established sole cause, with symptom persistence likely involving sustained immune remodeling, incomplete resolution of inflammatory signaling, and downstream neuroimmune disturbance (11). In addition to post-viral states, autoimmune activation, chronic low-grade metabolic inflammation, redox imbalance, and neuroimmune perturbation may also contribute to the chronic inflammatory milieu in ME/CFS (15).

Although their relative contribution may differ across patients, these inflammatory contexts may converge on downstream features such as mitochondrial stress, altered metabolite signaling, immune remodeling, and central fatigue manifestations. A concise overview of chronic inflammatory contexts relevant to ME/CFS is provided in **Table 2**.

**Table 2:** Chronic inflammatory contexts and metabolic remodeling pathways relevant to inflammation-associated fatigue.

Category	Factor/pathway	Representative relevance to fatigue biology	References
Inflammatory context	Post-infectious immune dysregulation	Plausible initiating context for sustained immune remodeling and fatigue	(13, 14)
	Long COVID/post-viral inflammatory state	Relevant because a subset of patients develop ME/CFS-like phenotypes	(2, 13, 14)
	Autoimmune activation	May contribute to persistent inflammatory signaling in selected patients	(11, 15, 102)
	Chronic low-grade metabolic inflammation	May amplify fatigue through immunometabolic stress and altered substrate handling	(15, 21, 23)
	Redox imbalance/neuroimmune perturbation	May contribute to mitochondrial stress, central fatigue, and symptom persistence	(10, 11, 15, 76)
Metabolic remodeling	Increased glycolytic reliance	Supports inflammatory activation but may reduce long-term bioenergetic efficiency	(18, 20, 25)
	TCA-cycle disruption/succinate accumulation	Links mitochondrial stress with inflammatory signaling	(21, 22, 24)
	Impaired fatty acid oxidation/lipid dysregulation	Associated with metabolic stress and altered immune-cell function	(23, 24, 105, 120)
	Mitochondrial dysfunction	Associated with reduced ATP production, ROS accumulation, and fatigue severity	(24, 37–39)
	Immune-cell metabolic reprogramming	Sustains chronic inflammation and may contribute to systemic energy imbalance	(18, 25, 103)

**Figure 1:**
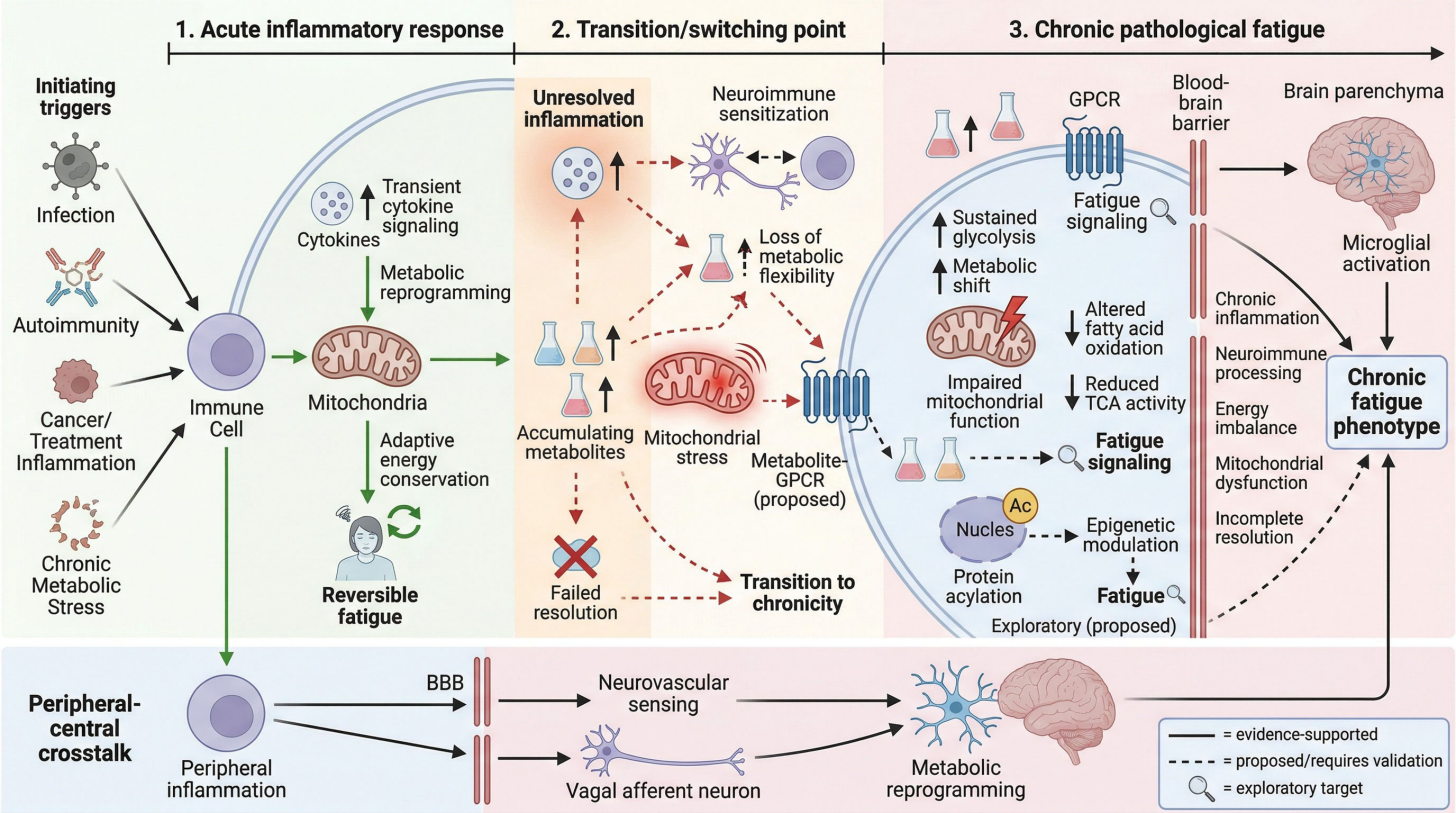
Stage-based framework of inflammation-driven fatigue. The figure illustrates progression from an acute adaptive sickness-response phase to chronic pathological fatigue. The transition or switching point is characterized by unresolved inflammation, metabolite accumulation, mitochondrial stress, neuroimmune sensitization, failed resolution, and loss of metabolic flexibility. In the chronic phase, immune-metabolic remodeling, mitochondrial dysfunction, peripheral-central signaling, microglial activation, and incomplete resolution converge on the chronic fatigue phenotype. Solid arrows indicate evidence-supported pathways, whereas dashed arrows indicate proposed or still-inferential links. Candidate therapeutic targets are shown as exploratory rather than established intervention points.

Given these multifaceted mechanisms, there is a compelling rationale to explore metabolic intermediates and signaling pathways as potential therapeutic targets. Interventions aimed at modulating GPCR signaling, correcting aberrant epigenetic regulation, or reprogramming immune-cell metabolism may represent mechanistically informed strategies for future evaluation in fatigue associated with chronic inflammatory states (16). Furthermore, identifying bioactive metabolites associated with fatigue biology, whether as candidate biomarkers or mechanistic mediators, may help inform future precision-medicine approaches (17). This comprehensive review will delve into the current understanding of cellular metabolism and bioactive metabolites in chronic inflammation-driven fatigue, emphasizing GPCR signaling, epigenetic acylation, immune metabolic remodeling, and their therapeutic implications. By integrating these dimensions, we aim to provide a holistic framework that advances both mechanistic insight and clinical translation in managing fatigue across chronic diseases. **Figure 1** presents an author-constructed, stage-based mechanistic framework synthesized from the literature reviewed here.

## 2 Methods

This review was conducted as a narrative, mechanism-oriented synthesis of the literature on chronic inflammation-driven fatigue, with particular emphasis on immunometabolism, bioactive metabolites, GPCR signaling, epigenetic acylation, and neuroimmune interactions. Literature retrieval was performed using major biomedical databases, including PubMed, Web of Science, and Scopus. The search strategy combined terms related to fatigue phenotypes, including chronic fatigue, ME/CFS, long COVID, cancer-related fatigue, and autoimmune fatigue, with terms related to inflammatory and metabolic mechanisms, including immunometabolism, metabolites, GPCR signaling, succinate, lactate, epigenetic acylation, histone acetylation, mitochondrial dysfunction, and microglia. Priority was given to peer-reviewed articles published in English, with emphasis on recent studies and influential mechanistic reports relevant to the scope of the review. Reference lists of key articles were also screened to identify additional relevant studies. We preferentially included studies addressing inflammatory, metabolic, neuroimmune, or therapeutic mechanisms related to fatigue, while excluding reports with limited relevance to the mechanistic focus of the review, non-peer-reviewed sources, and studies lacking sufficient methodological detail. As this is a narrative review rather than a formal systematic review or meta-analysis, study selection was guided by thematic relevance and conceptual contribution rather than by predefined quantitative synthesis criteria.

### 2.1 Mechanistic insights into chronic fatigue and immune metabolism

#### 2.1.1 Cellular metabolic reprogramming in chronic inflammation-driven fatigue

##### 2.1.1.1 Aberrant activation of metabolic pathways

Chronic inflammation fundamentally disrupts cellular metabolic homeostasis, leading to aberrant activation of key metabolic pathways such as glycolysis, fatty acid oxidation, and the tricarboxylic acid (TCA) cycle. Under physiological conditions, these pathways maintain a tightly regulated balance, ensuring sufficient ATP production and biosynthetic precursor supply. However, in chronic inflammatory states, persistent immune activation and the release of pro-inflammatory cytokines reprogram cellular metabolism, shifting energy production toward glycolysis even under normoxic conditions—a phenomenon reminiscent of the Warburg effect observed in cancer cells. For example, in autoimmune diseases such as rheumatoid arthritis (RA), T cells exhibit a metabolically reprogrammed state characterized by enhanced diversion of glucose into the pentose phosphate pathway and biosynthetic activity (18), while mitochondrial dysfunction and impaired oxidative phosphorylation further contribute to bioenergetic imbalance (19). This metabolic rewiring is not restricted to immune cells but extends to tissue-resident cells, which, under chronic inflammatory stress, experience a microenvironment rich in lactate and depleted in oxygen and glucose, resulting in impaired cellular mobility and function. Persistent activation of glycolytic flux, together with impaired mitochondrial ATP generation, may produce a state of bioenergetic insufficiency that plausibly contributes to fatigue in chronic inflammatory settings (20).

The imbalance among glycolysis, fatty acid oxidation, and the TCA cycle is further amplified by the dysregulation of lipid metabolism. In chronic inflammatory conditions such as obesity, diabetes, and cardiovascular disease (21), activation of the succinate–GPR91 axis has emerged as an important link between metabolic dysregulation, inflammatory signaling, and mitochondrial dysfunction (22). Succinate, a TCA cycle intermediate, accumulates under hypoxic and inflammatory conditions and acts as a signaling molecule through its receptor GPR91, promoting pro-inflammatory responses and exacerbating mitochondrial dysfunction. This not only accelerates tissue damage but also disrupts the normal flux of the TCA cycle, further impairing efficient ATP production. In parallel, fatty acid metabolism is often compromised in various chronic diseases, leading to abnormal lipid accumulation and further metabolic stress (23). The convergence of these metabolic derangements may generate a state of cellular energy stress that contributes to fatigue symptoms reported across chronic inflammatory disorders (24).

Metabolic reprogramming in chronic inflammation also extends to the immune compartment, where immune cells such as macrophages, T cells, and dendritic cells adapt their metabolic profiles to support sustained activation and effector functions. For instance, in the context of rheumatoid arthritis, metabolic mis-regulation is a pathogenic driver in all phases of disease progression, with mitochondrial insufficiency and altered glucose metabolism shaping the inflammatory microenvironment (25). Similarly, in metabolic diseases such as type 2 diabetes, the interplay between chronic low-grade inflammation and altered insulin signaling disrupts glucose and lipid homeostasis, reinforcing a vicious cycle of metabolic and immune dysfunction (23). Notably, the cGAS-STING pathway, activated by mitochondrial stress and energy imbalance, serves as a critical link between immune responses and metabolic disorders, further perpetuating inflammation and metabolic disruption (26). These findings suggest that the persistent metabolic reprogramming of immune cells not only sustains chronic inflammation but also directly impairs systemic energy metabolism, potentially explaining the overlap between inflammatory and metabolic symptoms such as fatigue.

The cumulative effect of these metabolic abnormalities is the emergence of energy supply obstacles, which are increasingly recognized as a central mechanism driving fatigue in chronic inflammatory diseases. Metabolomic studies in fatigue-associated disease contexts, including cancer-related fatigue and ME/CFS, have identified disturbances in glycolysis-, fatty-acid-, and TCA-cycle-related pathways, together with altered levels of metabolites such as lactate, succinate, and amino acids (27, 28). These metabolic signatures are distinct from those observed in non-fatigued individuals and are often accompanied by markers of oxidative stress and mitochondrial dysfunction. Sustained metabolic reprogramming in chronic inflammation may compromise ATP production while also promoting the accumulation of signaling-active metabolic byproducts (29), some of which may interfere with neuromuscular and cognitive function and thereby contribute to fatigue perception (30). This mechanistic insight underscores the importance of targeting metabolic pathways as a therapeutic strategy to alleviate fatigue and restore energy homeostasis in chronic inflammatory conditions. Key metabolic pathways and their corresponding impact on immune remodeling are summarized in **Table 2**.

##### 2.1.1.2 Inflammatory regulation by metabolites

Metabolites such as lactate and succinate have emerged as critical signaling molecules in the regulation and amplification of inflammatory responses. Traditionally viewed as mere byproducts of cellular metabolism, these molecules are now recognized for their capacity to modulate immune cell behavior and inflammatory gene expression. Lactate, generated in abundance during glycolysis, can function as a signaling metabolite that promotes pro-inflammatory responses in adipose tissue, in part by influencing macrophage polarization toward an inflammatory phenotype (31). Mechanistically, lactate directly interacts with prolyl hydroxylase domain-containing 2 (PHD2), stabilizing hypoxia-inducible factor 1-alpha (HIF-1α), and thereby upregulating the expression of interleukin-1β (IL-1β) in macrophages (32). In human adipose tissue, lactate levels have been reported to correlate positively with local inflammatory features independent of body mass index, supporting a role for lactate as more than a passive glycolytic byproduct (31). This evidence supports the notion that metabolites generated during altered metabolic states, such as obesity, serve not only as energy substrates but also as key amplifiers of immune activation.

Succinate, another TCA-cycle intermediate, is likewise implicated in the regulation and amplification of inflammatory responses (21). In activated macrophages, succinate accumulates and stabilizes HIF-1α, leading to increased transcription of pro-inflammatory cytokines, particularly IL-1β. Moreover, succinate can be sensed by the succinate receptor (SUCNR1), a GPCR expressed on immune and non-immune cells, further amplifying inflammatory signaling cascades. Recent work has highlighted that metabolite-sensing GPCRs, including receptors responsive to succinate and fatty-acid-related ligands, help coordinate the interplay between metabolic tissues and inflammatory signaling (22). This GPCR-mediated signaling axis is crucial in the context of metabolic diseases, where chronic low-grade inflammation is driven by aberrant metabolite signaling within adipose tissue and infiltrating immune cells (33). Given the centrality of succinate in both metabolic and immune pathways, targeting its signaling axis may provide therapeutic leverage in metabolic inflammation and associated fatigue.

Beyond lactate and succinate, a broader array of metabolites derived from both host and microbiota metabolism contribute to inflammatory regulation. Short-chain fatty acids (SCFAs) such as butyrate, propionate, and acetate, produced by gut microbiota fermentation of dietary fibers, have demonstrated potent immunomodulatory effects. SCFAs can suppress inflammatory cytokine production and promote regulatory T cell differentiation, thereby exerting anti-inflammatory effects in various tissues (12). Conversely, certain microbial metabolites, such as hippuric acid, have been shown to enhance pro-inflammatory responses by potentiating MyD88-dependent Toll-like receptor signaling and promoting M1 macrophage polarization (34). Taken together, these observations suggest that the inflammatory consequences of host- and microbiota-derived metabolites depend on their relative balance and on the broader dietary, microbial, and host context.

The regulatory influence of metabolites extends to the modulation of inflammatory cytokine expression, a process intimately linked to the development of fatigue in chronic inflammatory states. For instance, in the context of COVID-19, serum levels of agmatine and putrescine were found to be elevated and capable of activating the NF-κB pathway, resulting in heightened production of TNF-α and IL-6—cytokines closely associated with systemic inflammation and fatigue (35). Similarly, pyruvate, a central glycolytic metabolite, was identified as an inhibitor of TNF-α/NF-κB signaling, capable of attenuating colitis in animal models by targeting cytosolic phospholipase A2, highlighting the therapeutic potential of modulating metabolite signaling to control inflammation (36). These findings raise the possibility that metabolic interventions capable of altering the pool of bioactive metabolites may influence inflammatory cytokine profiles and, indirectly, fatigue-relevant biology in chronic inflammatory disease settings.

Collectively, the evidence underscores the central role of metabolites as both mediators and regulators of inflammation. Their ability to act through signaling pathways—such as GPCRs and transcriptional regulators like HIF-1α and NF-κB—positions them as critical nodes linking metabolic state to immune function. The dynamic interplay between metabolite production, immune cell activation, and cytokine expression not only shapes the trajectory of inflammation but also contributes to secondary symptoms such as fatigue. As research continues to unravel the complexity of metabolite-mediated signaling, it is anticipated that novel therapeutic strategies targeting these pathways may emerge, offering new hope for the management of chronic inflammation and its associated sequelae.

##### 2.1.1.3 Mitochondrial dysfunction and fatigue

Mitochondrial dysfunction has emerged as a central mechanism underlying fatigue in a variety of chronic inflammatory and systemic conditions. Under physiological conditions, mitochondria are responsible for generating the majority of cellular ATP through oxidative phosphorylation, thereby supporting the high energy demands of tissues such as skeletal muscle, immune cells, and the nervous system. However, chronic inflammatory states can precipitate mitochondrial damage through several converging pathways. Studies in Sjögren’s disease and ME/CFS have reported reduced mitochondrial oxygen consumption rate (OCR), ATP-linked respiration, and reserve capacity in patient immune cells, supporting the presence of impaired mitochondrial energy metabolism in these fatigue-associated conditions (37). Importantly, such mitochondrial abnormalities are unlikely to be restricted to immune cells alone, but may extend to skeletal muscle and other high-demand tissues, consistent with a broader state of systemic metabolic insufficiency relevant to persistent fatigue (38, 39). Reduced mitochondrial function has been reported to correlate with patient-reported fatigue severity, supporting an association between mitochondrial bioenergetic impairment and the subjective experience of fatigue (24).

Chronic inflammation may further exacerbate mitochondrial dysfunction by promoting reactive oxygen species (ROS) accumulation, thereby increasing oxidative damage to mitochondrial membranes, proteins, and DNA (40). Persistent immune activation and inflammatory cytokine signaling can generate a pro-oxidant intracellular environment that overwhelms antioxidant defenses and promotes structural mitochondrial injury, including disruption of cristae architecture and membrane potential (41). Such structural defects may reduce electron transport chain efficiency, decrease ATP synthesis, and increase electron leakage, thereby amplifying ROS production and contributing to a self-reinforcing cycle of cellular injury and bioenergetic failure (42). In preclinical systems and selected clinical contexts, interventions aimed at reducing oxidative stress or supporting mitochondrial function have shown mechanistic promise, although direct fatigue-focused evidence remains limited and heterogeneous (43).

The metabolic consequences of mitochondrial dysfunction are expected to be particularly relevant in tissues with high energetic demand (42). Skeletal muscle, for example, depends heavily on mitochondrial oxidative phosphorylation to sustain prolonged contractile activity. In chronic fatigue-related conditions, impaired mitochondrial respiration may contribute to reduced muscle endurance, altered lactate handling, and the earlier onset of fatigue during exertion. Metabolomic and bioenergetic analyses have suggested impaired substrate utilization together with abnormalities in respiratory-chain-related function, consistent with a mitochondrial bottleneck in energy metabolism (44). Similar patterns have been discussed in other chronic disease contexts, including cancer-related fatigue and post-viral syndromes, where mitochondrial impairment in peripheral and, potentially, central tissues may contribute to reduced physical and cognitive resilience (45, 46). It is plausible that convergent interactions between inflammatory signaling and mitochondrial bioenergetic failure represent one important shared mechanism contributing to fatigue across diverse disease contexts (10).

Beyond energy insufficiency, mitochondrial dysfunction also influences immune remodeling and systemic homeostasis. Damaged mitochondria can release mitokines and other signaling molecules that modulate immune responses and contribute to the persistence of chronic inflammation (47). The interaction among immune dysregulation, oxidative stress, and impaired mitochondrial turnover (mitophagy) may create a self-reinforcing loop in which ongoing inflammation further compromises mitochondrial quality control, thereby sustaining metabolic dysfunction and fatigue-related pathology (37). This reciprocal relationship suggests that strategies aimed at restoring mitochondrial health, through enhanced mitophagy, reduced oxidative stress, or support of mitochondrial biogenesis, deserve further evaluation as fatigue-relevant interventions in chronic inflammatory disease settings.

Taken together, evidence from clinical studies, animal models, and cellular analyses supports an important role for mitochondrial dysfunction—characterized by impaired ATP production, increased ROS accumulation, and disturbed mitochondrial turnover—in the biology of fatigue under chronic inflammatory conditions. The resulting metabolic insufficiency may reduce cellular energy availability while amplifying oxidative injury and immune dysregulation, thereby contributing to a self-perpetuating cycle associated with persistent fatigue.

#### 2.1.2 Bioactive metabolites and GPCR signaling in fatigue

##### 2.1.2.1 Key metabolite-mediated GPCR activation

A growing body of evidence underscores the central role of bioactive metabolites, such as SCFAs and fatty acid derivatives, in modulating immune and metabolic homeostasis via GPCRs. SCFAs, primarily produced by gut microbiota through fermentation of dietary fibers, serve as ligands for several GPCRs, including free fatty acid receptor 2 (FFA2/GPR43) and free fatty acid receptor 3 (FFA3/GPR41), which are highly expressed on immune cells and intestinal epithelium. The interaction between SCFAs and their cognate GPCRs orchestrates a range of physiological responses, from the regulation of gut hormone secretion (e.g., glucagon-like peptide-1 (GLP-1) release via FFA2) to the modulation of inflammatory pathways and energy metabolism (48, 49). This ligand-receptor specificity is not only crucial for maintaining mucosal immune tolerance and metabolic equilibrium but also represents a mechanism by which dietary and microbial cues are translated into host cellular responses. Notably, the spatial and temporal dynamics of GPCR activation by SCFAs—such as receptor internalization and compartmentalized signaling—can fine-tune downstream effects, suggesting that the physiological impact of SCFAs is context-dependent and may vary with cellular localization and metabolic state (48).

Beyond SCFAs, a diverse array of fatty acid derivatives and other metabolic intermediates, including hydroxycarboxylic acids, succinate, and eicosanoids, have been identified as endogenous GPCR agonists. For instance, hydroxycarboxylic acid receptors HCA2 and HCA3 are activated by metabolites such as β-hydroxybutyrate and kynurenic acid, respectively, and play pivotal roles in controlling lipolysis, modulating inflammatory responses, and influencing energy expenditure (33, 50). The succinate receptor SUCNR1 (GPR91) exemplifies the integration of metabolic stress signals with immune modulation, as succinate accumulation during inflammation can trigger SUCNR1-mediated signaling cascades that promote pro-inflammatory or tissue-protective responses depending on the cellular context (51). The physiological relevance of these metabolite-GPCR pairs is further highlighted by their tissue-specific expression patterns and ligand selectivity, which collectively enable precise regulation of metabolic and immune functions across different organ systems. This specificity suggests that metabolic cues can differentially shape immune cell behavior and tissue responses, depending on the repertoire of GPCRs expressed and the local metabolic microenvironment (33).

Recent advances in high-throughput screening technologies, such as PRESTO-Salsa and DCyFIR, have expanded our understanding of the metabolite-GPCR interactome. These platforms have enabled the systematic mapping of over a thousand human-associated metabolites against the entire GPCRome, revealing a previously underappreciated diversity of endogenous, exogenous, and microbiota-derived GPCR agonists (50, 52). Notably, microbiota-derived metabolites, including tryptamine, commendamide, and N-acyl amides, have been shown to selectively activate specific GPCRs, thereby influencing host immune responses, gut barrier integrity, and even neural signaling (53–55). These findings suggest an intricate network of inter-kingdom signaling, wherein microbial metabolism dynamically modulates host physiology through GPCR engagement. Consequently, it is conceivable that the composition of the gut microbiota and dietary patterns could be harnessed to selectively modulate GPCR-mediated pathways, potentially offering novel therapeutic avenues for metabolic and inflammatory diseases (52).

The physiological significance of metabolite-GPCR specificity is further underscored by the structural diversity of both receptors and ligands. Detailed structural studies have demonstrated that small polar metabolites, like succinate, engage solvent-exposed pockets on their cognate GPCRs, whereas lipid-derived ligands access membrane-embedded sites, often requiring integration into the lipid bilayer for efficient receptor activation (51). This structural complementarity underpins the exquisite selectivity observed in metabolite-GPCR pairing, ensuring that only specific metabolic cues elicit defined cellular responses. Furthermore, the existence of “biased signaling”—whereby different ligands or receptor conformations preferentially activate distinct downstream pathways—adds an additional layer of regulatory complexity, enabling the same GPCR to mediate context-dependent effects on metabolism and immunity (56). This paradigm of ligand-receptor specificity and signaling bias may explain why certain metabolites exert anti-inflammatory or pro-resolving functions through dedicated GPCRs, while others promote immune activation or metabolic adaptation.

Overall, the landscape of metabolite-mediated GPCR activation is characterized by a high degree of specificity in ligand-receptor pairing, which is intimately linked to the physiological roles of these pathways in immune regulation and metabolic homeostasis. The integration of dietary, microbial, and endogenous metabolic signals through GPCRs represents a central node in the maintenance of health and the pathogenesis of chronic inflammatory and metabolic diseases. Ongoing research into the molecular determinants of metabolite-GPCR interactions and their functional consequences is likely to yield new insights into therapeutic targeting of these pathways. However, the relevance of these metabolite-GPCR interactions to fatigue should presently be interpreted as biologically plausible rather than conclusively established. A comprehensive list of relevant bioactive metabolites, their GPCR receptors, and downstream effects on immune cells is provided in **Table 3**.

**Table 3:** Representative bioactive metabolites, relevant GPCRs, and reported immune effects.

Metabolite/ligand class	Relevant GPCR(s)	Reported immune/biological effect	References
Acetate, propionate, butyrate (SCFAs)	FFA2/GPR43, FFA3/GPR41	Modulate inflammatory signaling, Treg differentiation, and gut-immune homeostasis	(8, 12, 48)
β-Hydroxybutyrate	HCA2	Regulates lipolysis and inflammatory responses	(5, 33)
Kynurenic acid	HCA3	Linked to metabolic sensing and immunomodulatory signaling	(5, 50)
Succinate	SUCNR1/GPR91	Amplifies context-dependent inflammatory signaling and immune-metabolic responses	(22, 33, 51)
Microbiota-derived metabolites (e.g., tryptamine, commendamide, N-acyl amides)	Multiple GPCRs	Influence host immune responses, gut barrier function, and neural signaling	(52, 53, 55)
Metabolite-specific GPCR signaling	Multiple GPCRs	Ligand selectivity and biased signaling may shape context-dependent immune outcomes	(51, 56, 59, 60, 63)

##### 2.1.2.2 GPCR-mediated regulation of immune cell function

GPCRs are pivotal regulators of immune cell function, exerting broad influence over the metabolism and activity of macrophages, T cells, B cells, and other immune populations. Through their ability to sense a diverse array of extracellular ligands—including chemokines, lipids, metabolites, and even environmental cues like pH—GPCRs transduce signals that orchestrate immune cell migration, differentiation, activation, and effector functions. For instance, chemokine receptors, a major GPCR subfamily, direct leukocyte trafficking by interpreting chemokine gradients, thereby enabling immune surveillance and targeted responses to inflammation or infection (57). In the context of humoral immunity, GPCRs such as CXCR4 and CXCR5 guide B cell positioning within germinal centers, facilitating affinity maturation and antibody production (58). Importantly, the functional outcomes of GPCR signaling are highly context-dependent, with receptor subtype, ligand availability, and cellular environment all shaping downstream effects. Recent research has illuminated how metabolite-sensing GPCRs, such as GPR35 and GPR84, modulate immune cell metabolism and inflammatory responses, particularly in diseases characterized by chronic inflammation (59, 60). These findings suggest that the precise tuning of GPCR pathways is crucial for maintaining immune homeostasis, and that dysregulation can tip the balance toward pathological inflammation or immune suppression. Nevertheless, whether such GPCR-dependent immune and metabolic alterations translate directly into fatigue across distinct human disease settings remains unresolved.

GPCR signaling exerts intricate control over immune cell metabolism, which in turn influences cell fate decisions and functional phenotypes. For example, activation of certain GPCRs on macrophages can promote a metabolic shift toward glycolysis, supporting a pro-inflammatory (M1) phenotype, while alternative GPCR signaling may favor oxidative phosphorylation and an anti-inflammatory (M2) state (59). Similarly, in T cells, GPCRs such as sphingosine-1-phosphate receptors (S1PRs) and lysophosphatidylserine receptors regulate not only cellular trafficking but also metabolic reprogramming essential for T cell activation and differentiation (61, 62). The interplay between GPCR signaling and metabolic pathways is further exemplified by the role of GPCRs in sensing bioactive metabolites derived from the gut microbiota, which can modulate immune cell responses both locally and systemically (49, 63). Given the centrality of metabolic adaptation in immune cell function, it is plausible that chronic inflammatory states—characterized by persistent GPCR ligand availability and altered metabolic cues—may drive immune exhaustion and fatigue through sustained GPCR-mediated metabolic programming.

The link between GPCR-mediated immune signaling and fatigue symptoms has garnered increasing attention, particularly in the context of chronic inflammatory diseases and cancer. Emerging evidence suggests that aberrant GPCR signaling contributes to the persistence of inflammation and the development of immune cell dysfunction, both of which are implicated in fatigue pathogenesis (64). For instance, regulatory autoantibodies targeting GPCRs have been identified in conditions such as ME/CFS, where they are thought to modulate immune cell activity and perpetuate low-grade inflammation (64). Furthermore, GPCRs expressed in the gut-brain-immune axis have been shown to regulate autophagy and mitophagy via mechanistic target of rapamycin (mTOR) signaling, processes that are critical for cellular energy homeostasis and may influence the onset of fatigue through effects on mitochondrial function and immune cell viability (65). These observations point to a multifaceted role for GPCRs in bridging inflammatory signaling, cellular metabolism, and the subjective experience of fatigue, raising the possibility that interventions targeting specific GPCR pathways could alleviate fatigue by restoring immune and metabolic balance.

Recent advances have also highlighted the therapeutic potential of targeting GPCR signaling in immune cells to modulate inflammatory responses and ameliorate fatigue-related symptoms. Small-molecule modulators, monoclonal antibodies, and biased ligands that selectively influence GPCR activity are under investigation for their ability to recalibrate immune cell function without broadly suppressing immunity (66, 67). For example, antagonists of pro-inflammatory GPCRs such as GPR84 have demonstrated efficacy in reducing innate immune cell activation and migration in preclinical models of metabolic and inflammatory diseases (60). Conversely, agonists of metabolite-sensing GPCRs like GPR35 may enhance epithelial barrier function and exert anti-inflammatory effects, as shown in models of colitis (68). Given the heterogeneity of GPCR expression across immune cell subsets and disease states, precision targeting of GPCR pathways offers a promising strategy for mitigating chronic inflammation and its sequelae, including fatigue. It is conceivable that future therapies could leverage the cell-type and context-specific actions of GPCRs to reprogram immune metabolism and restore homeostasis in patients suffering from inflammation-driven fatigue.

Collectively, GPCR signaling constitutes a central regulatory axis in immune cell metabolism and function, with far-reaching implications for the pathogenesis of chronic inflammation and associated fatigue. The diverse repertoire of GPCRs enables immune cells to integrate a wide range of extracellular signals, coordinating metabolic and functional responses that are essential for effective immunity and tissue homeostasis. Dysregulation of GPCR pathways not only underpins immune dysfunction in chronic disease but also represents a tractable target for therapeutic intervention. Ongoing research into the cell- and context-specific roles of GPCRs will be crucial for the development of tailored strategies aimed at alleviating fatigue and restoring immune equilibrium in chronic inflammatory conditions, thereby dampening the systemic inflammatory signals that drive central and peripheral fatigue.

##### 2.1.2.3 GPCR signaling and neuro-immune interactions

GPCRs serve as crucial molecular bridges between metabolic signals and the nervous and immune systems, orchestrating complex responses that shape fatigue perception in chronic inflammatory conditions. A growing body of research highlights how bioactive metabolites, including neuropeptides and lipid mediators, engage GPCRs expressed on both neural and immune cells, thereby modulating neuroimmune communication and influencing behavioral states such as fatigue. For example, the chemokine CXCL13, through its receptor CXCR5—a canonical GPCR—has been implicated in the promotion of neuroimmune interactions and neuroinflammation within the sensory system, contributing to the pathogenesis of chronic pain and certain neurological diseases (69, 70). This CXCL13-CXCR5 axis not only recruits immune cells but also amplifies neuroinflammatory signaling, which can alter neural circuit function and potentially intensify fatigue sensation. The dual role of such chemokine-GPCR pathways in both immune modulation and neural sensitization suggests that targeted interventions at this interface may warrant further evaluation in fatigue states associated with chronic inflammation.

An additional layer of complexity in inflammation-associated fatigue lies in the metabolic crosstalk between the periphery and the central nervous system (CNS). Although peripheral immunometabolic remodeling is a major source of inflammatory and metabolic signals, fatigue is ultimately shaped within central neuroimmune circuits (65). Peripheral metabolites such as lactate and succinate may influence the CNS through several non-mutually exclusive routes, including transporter-mediated passage across the blood-brain barrier, signaling at the level of endothelial and perivascular cells, and indirect neural communication via vagal afferents (71). Through these pathways, peripheral metabolic stress may be translated into central alterations in glial activation, neurotransmission, and sickness-related behavioral programs (72). In particular, microglia are well positioned to integrate circulating inflammatory cues, metabolite-associated signals, and neural inputs, thereby linking peripheral immune activation to central changes in energy regulation, motivational state, and fatigue perception (73). Accordingly, the peripheral-central axis should be considered an important mechanistic interface through which systemic inflammation and metabolic disturbance may acquire symptom-level expression in chronic fatigue states. In this context, microglial metabolic reprogramming may represent one downstream mechanism linking peripheral inflammatory-metabolic disturbance to persistent neuroinflammation and central fatigue manifestations.

Microglia, as the resident immune cells of the central nervous system, are likely to play an important role in the neuroinflammatory dimension of fatigue. These cells respond dynamically to circulating inflammatory mediators, metabolic stress signals, and neural inputs, and can undergo metabolic reprogramming that reinforces pro-inflammatory or maladaptive activation states (74). In chronic inflammatory settings, microglial activation may contribute to altered cytokine signaling, synaptic dysfunction, and sustained neuroimmune sensitization, thereby linking peripheral immune-metabolic disturbance to central fatigue manifestations (75). Given their strategic position at the interface of immune surveillance, metabolic sensing, and neuroinflammatory signaling, microglia should be considered a plausible cellular mediator of fatigue-related CNS dysfunction.

In ME/CFS, neuroinflammation is increasingly discussed as a potential contributor to both fatigue persistence and cognitive symptom burden, although its precise cellular and molecular basis remains incompletely resolved (76). Available evidence from neuroimaging, neuroimmune, and systems-level studies suggests that sustained peripheral immune activation, altered metabolite-associated signaling, and glial reactivity may together promote a state of chronic central neuroimmune disturbance (77). Within this framework, altered cytokine signaling, microglial activation, and impaired metabolic support within the CNS may contribute to central fatigue perception, reduced cognitive endurance, and symptom chronicity (78). Neuroinflammation in ME/CFS should therefore be interpreted cautiously, not as an isolated CNS event, but as part of a broader peripheral-central inflammatory network.

Astrocytes also participate actively in neuroimmune crosstalk, and their responses are shaped by GPCR signaling together with local inflammatory and metabolic cues (79). Recent transcriptomic and mechanistic studies indicate that astrocytes can mount context-specific responses relevant to neuroinflammation, synaptic regulation, and metabolic support (80). Because astrocytes play central roles in maintaining neuronal homeostasis and in coordinating interactions with immune cells and microglia, it is plausible that astrocytic GPCR-dependent signaling represents an additional node through which metabolic, neural, and immune cues may converge in chronic fatigue-related states (81).

Whether ME/CFS should be regarded as a neurodegenerative condition remains unresolved. At present, the available literature more strongly supports a model of chronic neuroimmune disturbance, glial activation, and metabolic dysregulation within the CNS than one of established progressive neurodegeneration (76). Although persistent neuroinflammation and impaired neural metabolic support may contribute to cognitive dysfunction, central fatigue, and symptom chronicity, current evidence is insufficient to conclude that ME/CFS follows a classical neurodegenerative trajectory. Accordingly, caution is warranted when interpreting CNS abnormalities in ME/CFS, which may be better understood in terms of functional, neuroimmune, and bioenergetic disruption.

Several classes of bioactive peptides, such as calcitonin gene-related peptide (CGRP), adrenomedullin (AM), and their related family members, exemplify the direct influence of metabolites on neural circuits through GPCR engagement. These peptides activate class B GPCRs (e.g., calcitonin receptor-like receptor (CLR) and calcitonin receptor (CTR)) in complex with receptor activity-modifying proteins (RAMPs), initiating cyclic adenosine monophosphate (cAMP)-dependent signaling cascades that regulate pain perception, neuroimmune interactions, and vascular tone (82, 83). The duration and intensity of GPCR-mediated signaling by these peptides vary depending on their structural features, with some variants inducing prolonged cAMP responses that may sustain neuroimmune activation and contribute to persistent fatigue. The pharmacological manipulation of peptide-GPCR interactions—by harnessing long-acting peptide variants or selective antagonists—may, therefore, offer new strategies for attenuating chronic fatigue by dampening sustained neuroimmune signaling. These findings suggest that the temporal dynamics of GPCR signaling, shaped by the molecular characteristics of their ligands, play a pivotal role in modulating fatigue-related neural and immune responses.

The endocannabinoid system represents another axis through which metabolic signals modulate neuroimmune interactions via GPCRs. Endocannabinoids, acting primarily through cannabinoid receptor 1 (CB1) and cannabinoid receptor 2 (CB2), orchestrate a wide array of physiological processes including analgesia, mood regulation, and immune modulation (84). Beyond their classical receptors, endocannabinoid congeners can also activate other GPCRs, such as GPR55, and non-GPCR pathways, further broadening their influence over neuroimmune crosstalk. Endocannabinoid signaling is particularly important in the fine-tuning of synaptic strength, neuroprotection, and the resolution of neuroinflammation, processes that are frequently dysregulated in chronic inflammatory states associated with fatigue. The ability of endocannabinoids to dampen neuroinflammatory cascades and restore synaptic homeostasis positions them as potential modulators of fatigue sensation, especially in conditions where chronic inflammation perturbs neuroimmune balance.

Opioid peptides and their receptors provide a classic example of evolutionary conserved GPCR-mediated neuroimmune regulation, with direct implications for fatigue and sickness behavior. Endogenous opioids, such as enkephalins and endorphins, as well as their alkaloid analogs (e.g., morphine), interact with distinct GPCR subtypes (μ, δ, and κ opioid receptors) expressed on both neurons and immune cells (85). These interactions not only suppress nociception but also differentially modulate immune surveillance and inflammation, reflecting a sophisticated system of neuroimmune integration. The evolutionary elaboration of opioid receptor subtypes and their coupling to intracellular signaling pathways, such as nitric oxide synthase activation, highlights the adaptive significance of neuroimmune crosstalk in maintaining organismal homeostasis under stress and inflammation. Considering the broad distribution of opioid receptors and their ligands, it is reasonable to propose that opioid-GPCR signaling pathways contribute to the modulation of fatigue perception during chronic inflammatory states, potentially by influencing both central and peripheral immune responses.

These observations indicate that the intricate interplay between metabolites, GPCR signaling, and neuroimmune interactions forms a dynamic regulatory network that shapes fatigue perception in chronic inflammation. By targeting specific nodes within this network—including microglial and astrocytic signaling pathways, chemokine-GPCR axes, and peptide or endocannabinoid signaling—future therapeutic strategies may more effectively modulate neuroimmune dysfunction associated with chronic fatigue.

#### 2.1.3 Metabolite-mediated epigenetic acylation and immune regulation

##### 2.1.3.1 Acyl-coenzyme A derivatives and histone acylation

Although metabolite-mediated acylation has emerged as an important mechanism linking cellular metabolism to inflammatory gene regulation, its specific contribution to chronic fatigue remains incompletely defined. Much of the available evidence derives from oncology, sepsis, and broader immunometabolic research, and its relevance to fatigue should therefore be interpreted cautiously pending direct experimental validation in fatigue-related settings. Acyl-coenzyme A (acyl-CoA) derivatives, such as acetyl-CoA and butyryl-CoA, serve as critical acyl donors in the regulation of histone modifications, linking cellular metabolism to epigenetic control of gene expression. Histone acetyltransferases (HATs), such as KAT2A and HBO1, have been shown to possess the capacity to utilize a variety of acyl-CoA species—including acetyl-CoA, propionyl-CoA, butyryl-CoA, and succinyl-CoA—to install diverse acyl marks on histone lysine residues (86, 87). The structural basis for substrate selection by HATs involves the accommodation of different acyl chains within their catalytic domains, with the length and electrostatic properties of the acyl group influencing specificity and efficiency (86). Notably, HBO1 was identified as a versatile acyltransferase that can catalyze not only acetylation but also propionylation, butyrylation, and crotonylation, especially at transcriptional start sites of actively transcribed genes (87). These findings underscore the molecular adaptability of HATs in sensing and responding to fluctuations in intracellular acyl-CoA pools, suggesting that the metabolic state of a cell could dynamically shape the histone acylation landscape and, consequently, gene expression programs.

The impact of histone acylation on inflammation-related gene expression has become increasingly evident. Acetyl-CoA, produced through multiple metabolic pathways including glycolysis and fatty acid oxidation, is a well-established donor for histone acetylation, which is associated with an open chromatin state and active gene transcription (88, 89). Beyond acetylation, other short-chain acylations—such as propionylation, butyrylation, crotonylation, and succinylation—have been identified as functionally significant modifications that can mark histones in a metabolically sensitive manner (88). The abundance and nuclear availability of each acyl-CoA species are regulated by distinct metabolic pathways and nutrient sources, with compartmentalized synthesis being essential since acyl-CoAs cannot cross mitochondrial membranes directly (88, 90). For instance, propionyl-CoA derived from the catabolism of branched-chain amino acids, such as isoleucine, is enriched in the nucleus and has been linked to histone propionylation, demonstrating a direct metabolic-epigenetic crosstalk (90). This compartmentalization implies that local metabolic activity within the nucleus may exert a fine-tuned influence on chromatin structure and gene regulation, particularly under inflammatory conditions where metabolic fluxes are altered.

The functional consequences of histone acylation extend to the modulation of immune and stress responses. Genome-wide analyses have demonstrated that histone acylation marks, including acetylation, butyrylation, propionylation, and crotonylation, are dynamically regulated by nutrient availability and metabolic enzyme activity, with direct effects on gene expression profiles. For example, depletion of enzymes involved in acetyl-CoA synthesis—such as ATP citrate lyase or carnitine acetyltransferase—results in global loss of multiple acylation marks and significant changes in cellular metabolic states (91). Furthermore, specific acylations can act as immediate and reversible sensors of metabolic stress, enabling rapid cellular adaptation through transcriptional reprogramming. In inflammatory contexts, the availability of acyl-CoA donors and the activity of corresponding transferases may therefore determine the expression of key cytokines and regulators of immune cell function. It is plausible that manipulating these metabolic pathways could offer new strategies for modulating inflammation-driven fatigue and related pathologies by targeting the epigenetic machinery responsible for histone acylation.

Recent studies have also highlighted the complexity and diversity of acyl-CoA species and their roles in post-translational modification of histones. In addition to the well-studied acetyl-CoA, metabolites such as succinyl-CoA, crotonoyl-CoA, malonyl-CoA, and lactoyl-CoA have been shown to serve as donors for distinct lysine acylations, each conferring unique regulatory properties on chromatin and gene expression (88, 92). The relative abundance and reactivity of these acyl-CoAs, as well as the specificity of the modifying enzymes, contribute to the dynamic and context-dependent establishment of the histone acylation code. Notably, competitive and inhibitory interactions among different acyl-CoA species can modulate the activity of acyltransferases, suggesting that shifts in cellular metabolism—such as those induced by chronic inflammation—may lead to specific patterns of histone modification and altered transcriptional outputs (88). This intricate interplay between metabolism and the epigenome may underlie the persistent changes in gene expression observed in chronic inflammatory states and fatigue syndromes.

Thus, acyl-CoA derivatives—including acetyl-CoA, butyryl-CoA, and others—serve as essential metabolic intermediates that bridge cellular energy status with the epigenetic regulation of gene expression through histone acylation. The specificity and abundance of these modifications are determined by both the metabolic pathways generating acyl-CoAs and the molecular properties of the acyltransferases that install them. Through this metabolic-epigenetic axis, cells are able to rapidly adapt gene expression in response to environmental and nutritional cues, with significant implications for inflammation, immunity, and the persistence of fatigue symptoms. The specific roles of distinct acyl-CoA species in mediating histone modifications and immune regulation are detailed in **Table 4**.

**Table 4:** Acyl-CoA derivatives, histone/non-histone acylation, and immune regulation.

Metabolite/donor	Modification type	Reported relevance to immune or inflammatory regulation	References
Acetyl-CoA	Histone acetylation	Promotes transcriptionally active chromatin and inflammatory/metabolic gene regulation	(86, 88, 89)
Propionyl-CoA	Histone propionylation	Reflects metabolic-epigenetic coupling under altered nutrient states	(86, 90)
Butyryl-CoA	Histone butyrylation	Metabolically sensitive acyl mark with potential transcriptional relevance	(87, 88)
Crotonyl-CoA	Histone crotonylation	Associated with active chromatin and context-dependent gene regulation	(87, 88, 91, 138)
Succinyl-CoA	Histone/protein succinylation	Links TCA-cycle state with chromatin and protein regulation	(86, 88, 94, 96)
Lactate-related donor pool	Histone and non-histone lactylation	Linked to inflammatory transcriptional remodeling and macrophage functional adaptation	(92, 93, 95)
β-Hydroxybutyrate-related donor pool	β-Hydroxybutyrylation	Emerging metabolically sensitive modification relevant to immune regulation	(93, 94)
Non-histone acylation of regulatory proteins	Acetylation/succinylation/crotonylation	Can alter transcription factor activity, protein interactions, and immune-cell polarization	(94, 96, 97)

##### 2.1.3.2 Metabolite regulation of non-histone protein acylation

Metabolites serve as critical mediators of non-histone protein acylation, directly influencing the modification of transcription factors and signaling proteins, thereby regulating their function and stability. Recent advances in mass spectrometry have uncovered a diverse array of lysine acylations beyond acetylation—including crotonylation, lactylation, succinylation, and β-hydroxybutyrylation—on non-histone proteins, highlighting a complex regulatory network where metabolites act as both substrates and modulators of these modifications (93). For instance, the availability of specific acyl-CoA metabolites, which are products of central metabolic pathways, dictates the extent and specificity of protein acylation. This metabolic sensitivity is evident in the compartmentalization of acyl-CoA species, as their nuclear or cytosolic abundance can selectively influence the acylation of transcription factors and signal transducers, ultimately affecting gene expression and cellular adaptation to stress (88). As a result, the metabolic state of the cell is intricately linked to the acylation status of key regulatory proteins, suggesting that metabolic fluctuations may rapidly reprogram cellular signaling networks through dynamic acylation events.

Non-histone protein acylation has profound implications for immune cell polarization and function, as post-translational modifications (PTMs) such as succinylation, lactylation, and crotonylation modulate the activity, localization, and interaction of critical immune regulators (94). For example, in macrophages, increased glycolytic flux during inflammation leads to elevated lactate production and subsequent histone and non-histone lactylation, which in turn modulates the transcriptional response to inflammatory stimuli (95). Similarly, succinylation and crotonylation of metabolic enzymes and signaling proteins can shift immune cell polarization toward pro- or anti-inflammatory phenotypes, depending on the prevailing metabolic milieu. These modifications not only fine-tune the immune response but also provide a feedback mechanism whereby metabolic changes reinforce or resolve inflammatory states. Given the reversible nature of these acylations, targeting the enzymes responsible for their addition or removal—such as acyltransferases and deacylases—offers potential therapeutic avenues for modulating immune cell function in chronic inflammatory diseases (96). It is conceivable that metabolic interventions aimed at altering the availability of specific acyl donors could selectively reprogram immune cell phenotypes *in vivo*. However, whether such acylation-dependent immune reprogramming contributes directly to chronic fatigue phenotypes remains uncertain, as supporting evidence in fatigue-specific contexts is still limited.

The interplay between metabolite-driven acylation and non-histone protein function extends to the regulation of transcriptional activity and protein-protein interactions, with significant consequences for cellular homeostasis and disease progression. Acylation of transcription factors can alter their DNA-binding affinity, subcellular localization, and interaction with co-regulators, thereby orchestrating complex gene expression programs in response to metabolic cues. For instance, the acetylation of the transcriptional coactivator peroxisome proliferator-activated receptor gamma coactivator 1-alpha (PGC-1α) by GCN5 links cellular energy status to mitochondrial biogenesis and metabolic adaptation, underscoring the role of non-histone acylation in integrating metabolic and transcriptional networks (97). Moreover, the competitive dynamics among different acyl-CoA species for modification sites on proteins suggest that shifts in metabolite pools can rapidly redirect signaling outcomes, potentially contributing to immune cell plasticity and the resolution or perpetuation of inflammatory responses (88). This intricate crosstalk between metabolism and protein acylation highlights the importance of metabolic compartmentalization and enzyme specificity in fine-tuning cellular responses to environmental and pathological stimuli.

Emerging evidence also emphasizes the role of non-histone acylation in shaping the immune landscape during chronic inflammation. Novel acyl modifications, such as β-hydroxybutyrylation and malonylation, have been identified on key immune regulators, influencing processes ranging from cytokine production to antigen presentation (94). These modifications often act in concert with other PTMs, creating a multilayered regulatory system that integrates metabolic status with immune function. As research continues to unravel the functional consequences of these modifications, it becomes increasingly apparent that non-histone protein acylation serves not only as a molecular readout of metabolic flux but also as an active driver of immune cell fate decisions. The therapeutic manipulation of metabolic pathways to influence non-histone acylation thus represents a promising strategy for the treatment of inflammation-driven fatigue and related disorders.

In aggregate, metabolite-mediated non-histone acylation constitutes a dynamic interface between cellular metabolism and immune regulation, orchestrating the activity of transcription factors and signaling proteins in response to metabolic cues. These modifications play a pivotal role in immune cell polarization and function, offering novel insights into the metabolic control of inflammation and potential targets for therapeutic intervention. As our understanding of the specificity and reversibility of these acyl marks deepens, new opportunities arise for the precise modulation of immune responses in chronic inflammatory states.

##### 2.1.3.3 Epigenetic acylation and chronic fatigue phenotype

Epigenetic acylation modifications, including acetylation and other acylations of histone and non-histone proteins, have been proposed as potential regulators of gene expression programs relevant to chronic fatigue; however, this association remains largely inferential and has not yet been directly established in most fatigue-related contexts. During chronic inflammation, metabolic reprogramming in immune and non-immune cells leads to altered levels of key metabolites such as acetyl-CoA, nicotinamide adenine dinucleotide (NAD+), and S-adenosylmethionine, which serve as substrates or cofactors for epigenetic enzymes. These metabolic changes can directly impact the activity of HATs and deacetylases (HDACs), resulting in global or locus-specific shifts in chromatin accessibility and transcriptional output. For instance, in conditions of chronic inflammation, persistent metabolic stress can upregulate glycolytic flux and lipid metabolism, leading to an increased supply of acetyl-CoA and subsequent hyperacetylation of histones at promoters of pro-inflammatory genes, thereby sustaining the inflammatory milieu and contributing to fatigue-related gene expression profiles (98, 99). This close coupling of metabolism and epigenetic regulation suggests that metabolic intermediates act as both sensors and effectors of the inflammatory state, driving the persistence of fatigue symptoms.

The relevance of acylation modifications extends beyond immune cells to other cell types implicated in fatigue, such as endothelial cells and cells of the central nervous system. For example, metabolic reprogramming in endothelial cells during chronic inflammation not only alters their function but also modifies their epigenetic landscape, influencing the expression of adhesion molecules and cytokines that perpetuate immune cell recruitment and tissue inflammation (99). Similarly, in the central nervous system, glial cells undergo metabolic and epigenetic changes that shape neuroinflammatory responses and may contribute to the central component of chronic fatigue (100). The interplay between metabolic flux and epigenetic marks in these diverse cell types under chronic inflammatory conditions creates a feedback loop that reinforces the expression of fatigue-associated genes and the persistence of pathological fatigue phenotypes. Given the observed plasticity of these epigenetic modifications, it is plausible that interventions targeting metabolic pathways could have a profound impact on the epigenetic state and functional output of these cells, potentially alleviating fatigue.

Crucially, the dynamic nature of epigenetic acylation provides a promising avenue for therapeutic intervention in chronic fatigue. Unlike genetic mutations, epigenetic modifications are reversible, and their regulation by metabolic state means that small molecules targeting metabolic enzymes or epigenetic writers/erasers can modulate gene expression programs associated with fatigue. For example, targeting metabolic enzymes that regulate the availability of acetyl-CoA or NAD+ may indirectly influence histone acetylation and sirtuin activity, thereby reprogramming the inflammatory gene expression profiles that drive fatigue (98, 101). Moreover, the identification of key epigenetic regulators—such as histone deacetylases or methyltransferases—that are aberrantly activated in chronic inflammation opens the door to the development of selective inhibitors or activators as potential therapeutics. This strategy is supported by emerging evidence that metabolic and epigenetic interventions can restore immune cell homeostasis and resolve chronic inflammation in preclinical models (102).

The available evidence suggests that epigenetic acylation modifications act as a molecular bridge linking cellular metabolism to the gene expression programs that sustain chronic fatigue under inflammatory conditions. By targeting these modifications or their upstream metabolic regulators, it may be possible to rewire the transcriptional landscape of key cell types, offering new strategies for the treatment of fatigue in chronic inflammatory diseases. As research advances, the integration of multi-omics approaches to map the interplay between metabolism, epigenetics, and immune function will be vital for identifying robust biomarkers and therapeutic targets tailored to the unique metabolic-epigenetic signatures of chronic fatigue. Direct validation in patient-derived samples and fatigue-relevant experimental models will be necessary to determine whether these epigenetic alterations are mechanistically involved in fatigue pathogenesis or instead reflect broader inflammatory remodeling.

#### 2.1.4 Immune metabolic remodeling in chronic fatigue

##### 2.1.4.1 Alterations in immune cell energy metabolism

Under conditions of chronic inflammation, immune cells undergo profound metabolic reprogramming to meet the heightened energy demands associated with persistent activation and altered functional states. For instance, macrophages can polarize toward either a pro-inflammatory M1 phenotype or an anti-inflammatory M2 phenotype, each characterized by distinct metabolic signatures. M1 macrophages predominantly rely on glycolysis to rapidly generate ATP and biosynthetic intermediates, supporting their robust production of pro-inflammatory cytokines and ROS. In contrast, M2 macrophages favor oxidative phosphorylation and fatty acid oxidation, aligning with their roles in tissue repair and resolution of inflammation. Chronic inflammatory environments often disrupt this balance, leading to a predominance of M1-like metabolic programming, which perpetuates tissue damage and impairs resolution pathways (102). Similarly, T cell subsets such as T helper 17 (Th17) and regulatory T cells (Tregs) exhibit differential metabolic dependencies: Th17 cells are glycolysis-dependent, while Tregs utilize oxidative metabolism, particularly fatty acid oxidation, to sustain their suppressive functions. Disruption of this metabolic equilibrium under chronic inflammation can tilt the Th17/Treg balance toward a pro-inflammatory state, exacerbating immune-mediated pathology (103). In this context, the concept of metabolic flexibility is particularly relevant. Effective immune adaptation depends not only on the activation of individual pathways such as glycolysis or fatty acid oxidation, but also on the capacity to switch appropriately between substrates in response to functional demand and nutrient availability. Accordingly, increased reliance on fatty acid oxidation should not be interpreted uniformly as beneficial. In some chronic fatigue-related states, enhanced FAO may instead reflect impaired glucose oxidation or broader mitochondrial substrate inflexibility, indicating a compensatory yet metabolically constrained state rather than true restoration of energetic homeostasis. This distinction is particularly important when interpreting fatigue phenotypes, in which an apparent shift toward FAO may signify adaptive compensation in some settings but bioenergetic restriction in others.

The aberrant energy metabolism observed in immune cells during chronic inflammation is not merely a byproduct of activation but actively contributes to immune dysfunction and the pathogenesis of fatigue. As detailed in Section 2.1, immune cells in these hypoxic microenvironments undergo a metabolic switch similar to the Warburg effect, shifting dependency from oxidative phosphorylation to glycolysis. This metabolic switch is associated with increased lactate production, impaired mitochondrial function, and accumulation of metabolic byproducts that can directly suppress immune effector functions and promote cellular exhaustion (104). In such settings, immune cells exhibit reduced spare respiratory capacity and diminished ATP production, rendering them less capable of mounting effective responses to pathogens or resolving inflammation. This state of metabolic insufficiency is closely linked to the clinical manifestation of fatigue, as the immune system’s sustained energy consumption competes with other physiological systems, notably the central nervous system, for limited energetic resources (104). It is plausible that interventions aimed at restoring mitochondrial function or modulating glycolytic flux could ameliorate immune cell dysfunction and alleviate fatigue symptoms in chronic inflammatory states.

Beyond glucose metabolism, lipid metabolic pathways are increasingly recognized as critical regulators of immune cell fate and function during chronic inflammation. Recent studies have demonstrated that lipid uptake, *de novo* synthesis, and fatty acid oxidation are dynamically remodeled in myeloid and lymphoid cells in response to chronic inflammatory cues. Dysregulation of these pathways can lead to an accumulation of lipid intermediates, altered membrane composition, and the generation of bioactive lipid mediators that further modulate immune responses. For example, excessive lipid accumulation in macrophages can drive their polarization toward an immunosuppressive phenotype, while alterations in fatty acid metabolism in T cells can impair their proliferation and effector functions (105). These findings suggest that metabolic interventions targeting lipid pathways may hold therapeutic potential for restoring immune homeostasis and mitigating fatigue in chronic inflammation.

At the systems level, chronic inflammation induces a persistent reprogramming of both systemic and cellular energy metabolism. The immune system, when chronically activated, commandeers substantial amounts of energy-rich substrates, often at the expense of other organ systems such as the brain and skeletal muscle. This metabolic competition is thought to underlie many of the systemic symptoms observed in chronic inflammatory diseases, including fatigue, cognitive dysfunction, and muscle weakness (104). This metabolic rigidity not only impairs immune cell adaptability but also establishes a vicious cycle wherein energy deficits perpetuate immune dysfunction and systemic fatigue. These insights underscore the need for therapeutic strategies that target immune cell metabolism as a means of breaking the cycle of chronic inflammation and fatigue.

Viewed together, these findings indicate that chronic inflammation orchestrates complex and maladaptive changes in immune cell energy metabolism, driving shifts in metabolic phenotypes, impairing immune function, and promoting the development of fatigue. The interplay between metabolic reprogramming and immune cell dysfunction highlights the potential for metabolic interventions to restore immune competence and alleviate fatigue in chronic inflammatory diseases.

##### 2.1.4.2 Metabolite regulation of immune cell polarization

Metabolites such as lactate and succinate have emerged as pivotal regulators of immune cell polarization, particularly in the context of macrophage functional states. Lactate, a byproduct of glycolysis and a hallmark of the Warburg effect in tumor and inflamed tissues, can drive macrophage polarization toward the M2 phenotype, which is associated with anti-inflammatory functions and tissue remodeling. Studies in head and neck squamous cell carcinoma have demonstrated that tumor-secreted lactate induces M2 macrophage polarization, thereby suppressing inflammatory responses and adaptive Th1 immunity, and promoting angiogenesis and tissue remodeling (106). Similarly, in atherosclerosis, lactate and its epigenetic modification product, lactylation, have been shown to modulate not only macrophage polarization but also T cell differentiation and B cell metabolism, highlighting the complex dual roles of lactate in both promoting and resolving inflammation (107). These findings suggest that lactate is not merely a metabolic waste product but a key immunomodulatory molecule that can shape the immune landscape in chronic inflammatory conditions.

Succinate, a key intermediate in the TCA cycle, also exerts a significant influence on immune cell function. Elevated succinate levels can stabilize HIF-1α, promoting the expression of pro-inflammatory cytokines and supporting the polarization of macrophages toward the M1 phenotype, which is characterized by robust antimicrobial and pro-inflammatory activity (108). However, the regulatory effects of TCA cycle metabolites are context-dependent; for instance, α-ketoglutarate, another TCA cycle intermediate, has been reported to favor M2 polarization and exert anti-inflammatory effects (109). This metabolic flexibility underscores the intricate balance between pro- and anti-inflammatory states, which is tightly regulated by the availability and flux of key metabolites within the cellular microenvironment. Given the dynamic interplay between these metabolites, it is plausible that shifts in metabolic pathways during chronic inflammation may perpetuate immune cell dysfunction, thereby sustaining inflammation and contributing to fatigue.

The interaction between metabolites and immune cells extends beyond direct effects on polarization, encompassing broader crosstalk within the tissue microenvironment. For example, gut microbiota-derived metabolites, such as SCFAs and bile acids, have been shown to modulate macrophage phenotypes and influence systemic immune responses (110). In cardiovascular diseases, microbial metabolites like butyrate and trimethylamine-N-oxide (TMAO) regulate macrophage function, either attenuating or exacerbating inflammation and atherosclerotic progression (111). Furthermore, studies have highlighted the role of microbial metabolites such as hippuric acid in potentiating M1-like macrophage polarization via Toll-like receptor (TLR)-MyD88 signaling, linking gut microbial metabolism to innate immune activation and systemic inflammatory outcomes (34). These observations support the notion that metabolic-immune cell interactions are central to the persistence of inflammation and the development of chronic fatigue, especially in settings where the gut microbiome is disrupted or altered.

The sustained presence of specific metabolites in the microenvironment, resulting from metabolic reprogramming in immune and non-immune cells, can reinforce immune cell polarization patterns and drive chronic inflammatory states. For instance, in tumor microenvironments, the accumulation of lactic acid and other metabolites not only conditions macrophages toward an M2 phenotype but also suppresses cytotoxic T cell activity, facilitating immune escape and disease progression (112). Conversely, interventions that target metabolic pathways—such as the administration of probiotics or metabolic modulators—can reprogram immune cell metabolism and polarization. Probiotic-derived metabolites, for example, have been shown to shift tumor-associated macrophages from an M2 to an M1 phenotype, thereby enhancing anti-tumoral immunity and inhibiting cancer cell proliferation and migration (113). These findings imply that therapeutic strategies aiming to modulate metabolite levels or signaling could restore immune homeostasis and ameliorate chronic inflammation-induced fatigue.

Altogether, the regulation of immune cell polarization by metabolites like lactate and succinate is a multifaceted process involving direct metabolic signaling, epigenetic modifications, and extensive crosstalk within the microenvironment. The interplay between metabolite-driven immune cell polarization and the persistence of inflammation provides a mechanistic basis for the development and maintenance of fatigue in chronic inflammatory diseases. Targeting these metabolic-immune interactions holds promise for novel therapeutic interventions to resolve inflammation and improve clinical outcomes in fatigue-associated disorders.

##### 2.1.4.3 Immune metabolic remodeling and the inflammation-fatigue axis

Chronic inflammation is increasingly recognized as a central driver of persistent fatigue, a phenomenon often observed in conditions such as ME/CFS, post-acute sequelae of COVID-19 (long COVID), cancer, and autoimmune diseases. Immune-metabolic remodeling, characterized by altered energy substrate utilization and immune cell function, underpins the perpetuation of inflammation and the intensification of fatigue symptoms. In chronic inflammatory states, immune cells undergo metabolic reprogramming, shifting from oxidative phosphorylation to glycolysis, which not only sustains their effector functions but also leads to the consumption of energy-rich substrates at the expense of other organ systems, particularly the brain and muscles (104). This metabolic competition can result in systemic energy deficits, manifesting as profound fatigue, cognitive dysfunction, and muscle weakness. Moreover, the chronic activation of the immune system fosters a vicious cycle where metabolic dysfunction and inflammation reinforce each other, further disrupting energy homeostasis and exacerbating fatigue (114).

Several lines of evidence highlight how immune-metabolic remodeling contributes to the maintenance and amplification of the inflammation-fatigue axis. For instance, persistent low-grade inflammation, marked by elevated cytokines such as interleukin-6 (IL-6), interferon-gamma (IFN-γ), and tumor necrosis factor-alpha (TNF-α), has been linked to mitochondrial dysfunction in both immune and non-immune cells (115, 116). This dysfunction impairs ATP production and increases ROS, further fueling inflammatory responses and muscle catabolism. Notably, studies in ME/CFS and post-viral syndromes have demonstrated that immunosenescence—age-related immune decline—amplifies these maladaptive metabolic shifts, leading to cross-talk between immune, muscular, neuroendocrine, and vascular systems that sustains systemic fatigue (114, 117). It is plausible that interventions targeting metabolic pathways in immune cells may simultaneously dampen inflammation and restore energy balance, potentially alleviating fatigue in these chronic conditions.

Metabolic intermediates and bioactive metabolites, such as those derived from tryptophan (kynurenines), lipids, and amino acids, play crucial roles in modulating immune responses and fatigue. The kynurenine pathway, activated during inflammation, diverts tryptophan away from serotonin synthesis toward the production of immunomodulatory and neuroactive metabolites, some of which promote muscle catabolism and central fatigue (118, 119). Lipidomic studies in ME/CFS have revealed sex-specific alterations in phospholipids, ceramides, and oxylipins, correlating with the severity of fatigue and pain, and implicating disrupted lipid metabolism in immune dysfunction and symptom persistence (120). These findings suggest that the metabolic remodeling of immune cells not only sustains inflammation but also generates metabolites that directly influence fatigue pathogenesis and symptomatology.

The therapeutic landscape targeting immune metabolism in the context of inflammation-driven fatigue is expanding. Interventions that modulate metabolic pathways—such as the inhibition of indoleamine-2,3-dioxygenase (IDO) in the kynurenine pathway, supplementation with antioxidants (e.g., vitamin C), and approaches to restore mitochondrial function—have shown promise in preclinical and early clinical studies (118, 121). For example, IDO inhibition partially ameliorated inflammation in cancer models, though without fully preserving muscle mass, highlighting the complexity of metabolic-immune interactions (118). High-dose intravenous vitamin C has demonstrated anti-inflammatory and antioxidative effects, improving fatigue and neuropsychiatric symptoms in various chronic inflammatory diseases, including long COVID and cancer (121). Additionally, nutritional strategies, such as supplementation with amino acids like cystine and glutamine, have been shown to enhance fatty acid utilization, reduce perceived fatigue, and modulate immune responses during endurance exercise (122). Given the interplay between immune metabolism and the gut microbiome, probiotics and dietary interventions are also being explored for their capacity to restore metabolic and immune homeostasis, potentially mitigating fatigue (123).

This body of evidence supports immune metabolic remodeling as a pivotal mechanism linking chronic inflammation to the perpetuation and exacerbation of fatigue. The bidirectional relationship between immune cell metabolism and systemic energy homeostasis creates a self-sustaining axis that underlies the clinical manifestation of persistent fatigue. Targeted interventions aimed at modulating immune metabolism represent mechanistically relevant approaches for future evaluation in inflammation-associated fatigue. Ongoing research into the specific metabolic signatures and pathways involved will be essential for the development of personalized and effective therapies.

##### 2.1.4.4 Candidate biomarkers in inflammatory fatigue states

The identification of biomarkers for ME/CFS and related inflammatory fatigue states remains an important but unresolved challenge, as no single biomarker has yet shown sufficient consistency, specificity, and clinical validation for routine diagnostic application (124). Current evidence instead favors a multi-domain biomarker framework that integrates inflammatory, metabolic, neuroimmune, and bioenergetic features rather than relying on a single validated marker (125). Candidate biomarkers reported in ME/CFS include inflammatory mediators, metabolic intermediates, lipidomic and amino-acid-related signatures, mitochondrial and bioenergetic indices, and neuroimmune-associated factors measurable in peripheral biofluids (126). In particular, peripheral biofluid markers linked to neuroinflammatory signaling may help connect systemic immune dysregulation with central fatigue manifestations, although their reproducibility, disease specificity, and clinical utility remain insufficiently established (127, 128). A concise overview of representative biomarker categories relevant to ME/CFS is provided in **Table 5**.

**Table 5:** Candidate biomarker domains and exploratory therapeutic implications in inflammatory fatigue states.

Domain	Representative markers/targets	Reported relevance	References
Inflammatory biomarkers	IL-6, TNF-α, IFN-γ, chemokines	Frequently altered in subsets of patients, but not individually diagnostic	(15, 116, 128, 167–169)
Metabolomic biomarkers	Lactate, succinate, kynurenine, amino-acid and TCA-related signatures	Support the concept of immunometabolic disturbance and altered substrate utilization	(4, 24, 119, 129–132)
Lipidomic/bioenergetic biomarkers	Phospholipids, ceramides, oxylipins, mitochondrial indices	Relevant to fatigue severity, pain, and systemic energetic dysfunction	(24, 37–39, 120, 128, 168)
Neuroimmune-linked biomarkers	Peripheral biofluid markers linked to neuroinflammatory signaling	Mechanistically relevant but not yet sufficiently specific for clinical use	(76–78, 169)
Metabolites as therapeutic targets	Lactate, SCFAs and related metabolic pathways	May be useful for patient stratification and treatment-response monitoring	(133–137)
GPCR-targeted therapeutic axis	FFA2, FFA3, GPR81, GPR84 and related signaling pathways	Mechanistically attractive, but fatigue-specific clinical evidence remains limited	(33, 56, 59, 60, 142–152)
Metabolic/redox-targeted interventions	NAD+ support, antioxidants, mitochondrial/metabolic modulators	Biologically plausible, with preliminary or context-dependent evidence	(153, 154, 164–166)
Epigenetic-metabolic intervention axis	Metabolite-sensitive epigenetic pathways	Strong mechanistic rationale, but fatigue-specific translational validation remains limited	(138–140, 159, 161, 163)

#### 2.1.5 Therapeutic targets and novel intervention strategies

##### 2.1.5.1 Metabolites as biomarkers and therapeutic targets

The therapeutic strategies discussed in this section should be interpreted within an evolving translational landscape. While many of these approaches are supported by compelling mechanistic rationale and preclinical findings, direct clinical evidence for efficacy in fatigue remains limited. Accordingly, it is important to distinguish putative therapeutic targets from interventions that have been validated in fatigue-focused clinical settings. Metabolites such as lactate and SCFAs have attracted increasing interest as candidate biomarkers for fatigue-related phenotyping and therapeutic monitoring (129). Advances in metabolomics have enabled the identification of metabolite patterns associated with fatigue phenotypes, providing a potentially useful research framework for patient stratification and treatment-response assessment, although their clinical applicability remains to be established (130).

Elevated lactate has been linked to altered glycolytic metabolism and may be relevant to both peripheral and central components of fatigue; because it is measurable in accessible biofluids, it has been discussed as a candidate non-invasive fatigue-related metabolite (131, 132). Similarly, SCFAs have emerged as informative indicators of gut microbial, metabolic, and immune status, and altered SCFA-related profiles have been associated with inflammation-linked fatigue in selected disease contexts (133). The dynamic changes of these metabolites in response to interventions, such as dietary modifications or exercise regimens, further support their utility in monitoring therapeutic responses and guiding personalized management strategies.

The development of targeted approaches aimed at modulating key metabolites has also progressed. Pharmacological and nutritional interventions that influence lactate metabolism may affect energy homeostasis and acid–base balance, but direct evidence for fatigue-specific benefit remains limited (134). Manipulation of SCFA production through prebiotic, probiotic, or dietary-fiber-based strategies has shown immunoregulatory and metabolic effects, providing a mechanistic rationale for investigating these interventions in inflammation-associated fatigue (135, 136). Drug development efforts have also increasingly focused on metabolite-sensing GPCRs, which act as transducers of extracellular metabolic cues. Receptors such as FFAR2, FFAR3, and GPR81 have been implicated in the regulation of immune activation, inflammation resolution, and energy metabolism, making them mechanistically relevant candidates for future therapeutic investigation in fatigue-related disorders (33, 56, 137).

Recent research has also highlighted the role of metabolite-mediated epigenetic modifications, such as histone lactylation and crotonylation, in regulating gene-expression programs relevant to immune responses and cellular energy metabolism (138, 139). These post-translational modifications, which are linked to intracellular metabolite availability, provide an additional layer of metabolic control over inflammation-related biology. In preclinical settings, inhibitors targeting histone deacetylases or metabolic enzymes involved in lactate- and SCFA-related pathways have been shown to modulate inflammatory and metabolic gene-expression programs relevant to fatigue biology (140). The integration of metabolomics with machine-learning approaches has further supported the exploration of metabolite panels as candidate diagnostic or prognostic tools. Multi-omics studies suggest that metabolite panels incorporating lactate, SCFAs, and other intermediary metabolites may help distinguish fatigue-related states and inform response modeling, although robust external validation and disease-specific performance testing remain necessary (141).

Overall, metabolite-centered approaches remain promising from a mechanistic and biomarker-development perspective, but further fatigue-oriented validation is required before they can be translated into clinically robust strategies.

##### 2.1.5.2 GPCR-targeted drugs and metabolic modulators

GPCRs constitute one of the most extensively exploited drug-target families, with a substantial proportion of approved drugs acting directly or indirectly through GPCR-related mechanisms (142, 143). In the context of chronic inflammation-driven fatigue, the therapeutic potential of both GPCR antagonists and agonists is increasingly recognized due to their central roles in metabolic regulation, immune modulation, and neuroinflammation (59, 143). Recent advances in GPCR structural biology, especially through cryo-electron microscopy, have expanded understanding of ligand-binding modes and facilitated the development of pathway-selective, or biased, ligands (144, 145). For instance, the design of biased agonists or antagonists allows for selective engagement of G protein- or β-arrestin-mediated pathways, which may provide a framework for more selective modulation of metabolic and inflammatory pathways relevant to fatigue biology (56, 146). These advances have expanded the development of GPCR-targeting compounds involved in energy homeostasis, immune trafficking, and cytokine signaling, raising the possibility that more precise pathway-oriented interventions may eventually be applicable to inflammation-associated fatigue (67, 147). It is plausible that incorporation of GPCR-biased signaling principles into drug discovery may help identify compounds capable of more selectively modulating metabolic and immune dysregulation relevant to fatigue. GPCR-targeted drug development is further enriched by the discovery and validation of novel GPCRs implicated in metabolic and inflammatory diseases, such as orphan receptors GPR65, GPR18, and GPR84, which have emerged as promising targets for modulating immune cell-liver crosstalk and metabolic homeostasis (56). Modulation of orphan GPCRs such as GPR65, GPR18, and GPR84 has shown preclinical potential in inflammatory and metabolic disease models by influencing immune-cell infiltration and cytokine signaling (148). Similarly, modulation of free fatty acid receptors such as FFA2 and FFA3 is under active investigation for roles in adipose metabolism, glucose homeostasis, and inflammatory regulation, with synthetic ligands and allosteric modulators being evaluated mainly in preclinical and selected early translational settings (149, 150). The expanding pharmacological repertoire includes both small molecules and biologics, such as peptide agonists and allosteric modulators, designed to exploit the structural diversity and signaling specificity of GPCRs (151, 152). As research continues to elucidate the tissue-specific functions and downstream pathways of distinct GPCRs, the therapeutic landscape is likely to broaden, potentially enabling the development of personalized interventions for chronic fatigue syndromes associated with metabolic and immune dysfunction.

In parallel, metabolic modulators such as NAD+ supplements and antioxidants are gaining traction as adjunctive or stand-alone therapies in the management of chronic inflammation and fatigue. NAD+ is a central coenzyme in redox and energy metabolism, and reduced NAD+-related metabolic resilience has been associated with mitochondrial dysfunction, oxidative stress, and inflammatory activation—processes relevant to fatigue biology (153). Clinical studies have begun to evaluate NAD+-related supplementation for metabolic and inflammatory outcomes, while evidence for fatigue-specific benefit remains preliminary and condition-dependent (154). Antioxidants, by mitigating reactive oxygen species and restoring redox balance, also contribute to the normalization of cellular metabolism and may synergize with GPCR-targeted therapies to further dampen chronic inflammation and fatigue. Given that many metabolic intermediates and their receptors (SCFA-GPCR axes) are intimately involved in immune regulation and energy homeostasis, it is reasonable to consider that a combination of metabolic modulators and GPCR-directed agents could provide a more comprehensive approach to fatigue management.

Clinical research into GPCR-targeted drugs and metabolic regulators has yielded encouraging findings in several metabolic and inflammatory diseases, with some agents already approved or in late-stage clinical development for indications such as type 2 diabetes, obesity, nonalcoholic fatty liver disease (NAFLD), and neuroinflammation (142, 155, 156). However, direct clinical evidence for fatigue-specific benefit remains limited. For example, GLP-1 receptor agonists are well established in metabolic disease and also exhibit anti-inflammatory properties, although their relevance to fatigue outcomes remains to be determined (147). Similarly, antagonists of receptors such as GPR17 and cannabinoid receptor 1 (CB1R) are under investigation for their effects on energy balance, inflammatory signaling, and metabolic outcomes in obesity and related disorders (157, 158). Taken together, these advances support the view that GPCR-targeted and metabolically oriented interventions represent promising but still emerging candidates for chronic inflammation-driven fatigue, rather than clinically established fatigue therapies.

Overall, the convergence of GPCR-targeted pharmacology and metabolic modulation offers a fertile ground for innovative therapeutic strategies in chronic inflammatory fatigue. By leveraging advances in structural biology, ligand design, and systems pharmacology, future research is poised to deliver more selective, efficacious, and safer interventions that address both the metabolic and immune underpinnings of fatigue syndromes. These developments hold promise not only for symptom relief but also for modifying disease trajectories and improving quality of life for affected individuals.

##### 2.1.5.3 Prospects for epigenetic acylation modulators

The landscape of epigenetic therapeutics has rapidly expanded with the development of histone deacetylase (HDAC) inhibitors and acetyltransferase modulators, offering promising avenues for the regulation of gene expression in chronic inflammatory diseases and related fatigue syndromes. HDAC inhibitors, such as the novel oral Class I-targeting agent bocodepsin (OKI-179), have demonstrated manageable safety profiles and preliminary efficacy in advanced solid tumors, with evidence of increased histone acetylation in circulating T cells at pharmacologically relevant exposures (159). This supports the therapeutic potential of HDAC inhibition in modulating immune cell function and gene expression, which may be particularly relevant in the context of chronic inflammation-driven fatigue. Moreover, selective targeting of HDACs has shown protective effects in neurodegenerative disease models; for example, HDAC inhibitors ameliorated oxidative stress and neuroinflammation in Parkinson’s disease models, suggesting a broader applicability of these agents beyond oncology (160).

In addition to HDAC inhibitors, the modulation of HATs and related acetylation enzymes is gaining traction as a strategy to influence epigenetic landscapes. The enzyme KAT8, for instance, is critical for acetylation of histone H4 lysine 16 and is implicated in chromatin remodeling, gene expression, and immune responses (124). Targeting such enzymes could provide a means to fine-tune transcriptional programs involved in inflammation and cellular metabolism. Recent advances in CRISPR/dCas9-mediated epigenome editing further highlight the feasibility of locus-specific acetylation or deacetylation, enabling precise regulation of disease-relevant genes and secondary metabolic pathways (125). The application of such targeted epigenetic editing systems could be extended to immune or metabolic genes implicated in inflammation-driven fatigue, potentially offering higher specificity and fewer off-target effects compared to systemic inhibitors.

The interplay between metabolic regulation and epigenetic modification forms the foundation for emerging combination therapies. Acetyl-CoA, a central metabolic intermediate, serves as the substrate for histone acetylation, directly linking cellular metabolic state to chromatin dynamics (126). Inflammatory stimuli can modulate the activity of key enzymes such as pyruvate dehydrogenase (PDH) and ATP-citrate lyase (ACLY), thereby altering nuclear acetyl-CoA pools and influencing histone acetylation patterns (127, 161). Such findings suggest that interventions targeting metabolic pathways—either alone or in conjunction with epigenetic drugs—may synergistically restore immune homeostasis and alleviate chronic fatigue. For example, synchronous inhibition of acetyltransferases and metabolic kinases has been shown to sensitize resistant cancer cells to therapy, indicating that similar strategies might enhance the efficacy of anti-inflammatory or immunomodulatory treatments (162). Given the dynamic crosstalk between metabolism and epigenetic regulation, combining metabolic modulators with HDAC or HAT inhibitors could lead to more robust and durable therapeutic outcomes in inflammation-associated fatigue syndromes.

Natural products and dietary interventions also offer a complementary approach by modulating inflammatory, metabolic, and epigenetic pathways relevant to fatigue biology. Phytochemicals and short-chain fatty acids (SCFAs) derived from diet have been shown to regulate methylation and acetylation states of histones and DNA, thereby modulating inflammatory gene expression and metabolic function (163). Such compounds may serve as adjuncts to pharmacological epigenetic therapies, providing a multi-layered strategy to combat chronic inflammation and its sequelae, including fatigue. The integration of dietary, metabolic, and epigenetic interventions could thus represent a holistic approach to disease management, especially in the context of aging-related inflammation.

Several natural products and nutritionally derived bioactive compounds have been explored as candidate symptom-modifying interventions in ME/CFS and related fatigue states, primarily through proposed effects on oxidative stress, mitochondrial bioenergetics, inflammatory signaling, and metabolic resilience. Rather than acting through a single pathway, these agents are generally discussed as multimodal interventions that may influence symptom burden by improving cellular energy handling, attenuating redox imbalance, and reducing inflammation-associated physiological stress. For example, molecular hydrogen has been investigated for potential anti-fatigue effects in human and exercise-related settings, where its antioxidant and anti-inflammatory properties have been proposed as contributing mechanisms (164, 165). Red ginseng extract has likewise been associated with improved skeletal muscle energy metabolism and mitochondrial function in experimental fatigue models (166). In addition, amino acid-based nutritional support, including cystine/glutamine supplementation, has been linked to reduced perceived fatigue and enhanced fatty acid utilization in exercise-related fatigue settings (122). Taken together, these findings suggest that selected natural or nutrition-derived interventions may influence symptom domains relevant to fatigue; however, robust fatigue-specific clinical validation, particularly in ME/CFS, remains limited.

From a translational perspective, modulators of epigenetic acylation-related pathways—including HDAC inhibitors, HAT-related strategies, and diet-associated epigenetic agents—represent mechanistically relevant intervention directions in inflammation-associated fatigue. However, the current evidence base remains heterogeneous, with much of the support deriving from preclinical, mechanistic, or adjacent-disease literature rather than from fatigue-specific clinical trials. Future studies should therefore prioritize safety, specificity, and fatigue-oriented validation in clinically well-characterized patient populations. Current therapeutic targets and novel intervention strategies discussed in this review are consolidated in **Table 5**.

## 3 Conclusion

Chronic inflammation-driven fatigue represents a multifaceted pathological process characterized by intricate metabolic reprogramming at the cellular level and the regulatory influence of bioactive metabolites. Current evidence highlights GPCR signaling, epigenetic acylation-related regulation, and immune-metabolic remodeling as interconnected contributors to the inflammation-fatigue axis. These interconnected mechanisms not only contribute to the pathophysiology of fatigue but also highlight critical nodes for potential therapeutic intervention.

Chronic inflammation-associated fatigue emerges from dynamic interactions among immune activation, metabolic reprogramming, and epigenetic regulation. GPCRs serve as pivotal molecular conduits translating extracellular inflammatory cues into intracellular responses, thereby modulating immune cell function and metabolic states. Concurrently, epigenetic acylation modifications, including acetylation and other metabolite-sensitive acyl marks offer a reversible and finely tunable layer of gene expression control that adapts cellular metabolism and immune responses to chronic inflammatory stimuli. The metabolic remodeling within immune cells further amplifies this process, as key metabolites act as both substrates and signaling molecules, perpetuating fatigue-related pathways.

Importantly, the identification of specific bioactive metabolites as signaling entities extends beyond mere biomarkers; these molecules embody promising therapeutic targets. Modulating the activity of GPCRs, influencing epigenetic acylation patterns, or altering metabolite levels could disrupt the vicious cycle of inflammation and fatigue. This therapeutic potential is especially significant given the current paucity of effective treatments for chronic inflammation-induced fatigue, which severely impacts patient quality of life.

Balancing the diverse research perspectives reveals a nuanced landscape wherein metabolic, immunologic, and epigenetic domains converge. While some studies emphasize the primacy of immune metabolic remodeling, others highlight epigenetic regulation or receptor signaling as predominant factors. A comprehensive synthesis suggests that these pathways are not mutually exclusive but rather operate synergistically to drive fatigue pathology. This integrative understanding advocates for multi-targeted therapeutic strategies rather than isolated interventions.

Looking forward, the advancement of multi-omics technologies offers an unprecedented opportunity to dissect the complex interdependencies among metabolism, immunity, and epigenetics in chronic inflammation-induced fatigue. Integrative analyses combining genomics, epigenomics, metabolomics, and immunophenotyping are essential to unveil the precise molecular crosstalk and identify patient-specific biomarkers. Such insights will be instrumental in propelling the field toward precision medicine approaches, tailoring interventions to individual metabolic and immunological profiles.

Several limitations of this review should be acknowledged. First, the literature discussed here spans heterogeneous clinical entities, including ME/CFS, long COVID, cancer-related fatigue, and autoimmune-associated fatigue, which share partially overlapping but non-identical biological substrates. Second, the strength of evidence is uneven across topics: some mechanistic sections are supported by direct data from fatigue-related settings, whereas others are inferred from adjacent fields such as oncology, sepsis, neuroinflammation, or general immunometabolism. Third, translational interpretation remains constrained by the limited number of fatigue-focused clinical studies, particularly those integrating mechanistic biomarkers with symptom-defined outcomes. Accordingly, caution is warranted when extrapolating from mechanistic plausibility to fatigue-specific therapeutic relevance. These considerations underscore the need for future work that more directly links molecular mechanisms to clinically characterized fatigue phenotypes.

In conclusion, the evolving landscape of chronic inflammation-driven fatigue research highlights a sophisticated network of GPCR signaling, epigenetic acetylation, and immune metabolic remodeling. These interconnected mechanisms not only deepen our understanding of fatigue pathogenesis but also pave the way for innovative diagnostic and therapeutic modalities. Future endeavors must prioritize integrative, multi-omics research frameworks and translational studies to bridge mechanistic insights with clinical application, ultimately improving outcomes for patients burdened by this debilitating condition.

## References

[1] Nikitina MA Bragina EY Ivanova SA Boyko AS Levchuk LA Nazarenko MS . Association of inflammation and chronic fatigue syndrome in patients with Parkinson’s disease. Zh Nevrol Psikhiatr Im S S Korsakova. (2024) 124:79–87. doi: 10.17116/jnevro202412409179. PMID: 39435781

[2] Saito S Shahbaz S Osman M Redmond D Bozorgmehr N Rosychuk RJ . Diverse immunological dysregulation, chronic inflammation, and impaired erythropoiesis in long COVID patients with chronic fatigue syndrome. J Autoimmun. (2024) 147:103267. doi: 10.1016/j.jaut.2024.103267. PMID: 38797051

[3] Che X Ranjan A Guo C Zhang K Goldsmith R Levine S . Heightened innate immunity may trigger chronic inflammation, fatigue and post-exertional malaise in ME/CFS. NPJ Metab Health Dis. (2025) 3:34. doi: 10.1038/s44324-025-00079-w. PMID: 40903540 PMC12408823

[4] Al-Hakeim HK Khairi Abed A Rouf Moustafa S Almulla AF Maes M . Tryptophan catabolites, inflammation, and insulin resistance as determinants of chronic fatigue syndrome and affective symptoms in long COVID. Front Mol Neurosci. (2023) 16:1194769. doi: 10.3389/fnmol.2023.1194769. PMID: 37333619 PMC10272345

[5] Husted AS Trauelsen M Rudenko O Hjorth SA Schwartz TW . GPCR-mediated signaling of metabolites. Cell Metab. (2017) 25:777–96. doi: 10.1016/j.cmet.2017.03.008. PMID: 28380372

[6] Sabari BR Zhang D Allis CD Zhao Y . Metabolic regulation of gene expression through histone acylations. Nat Rev Mol Cell Biol. (2017) 18:90–101. doi: 10.1038/nrm.2016.140. PMID: 27924077 PMC5320945

[7] Maya J . Surveying the metabolic and dysfunctional profiles of T cells and NK cells in myalgic encephalomyelitis/chronic fatigue syndrome. Int J Mol Sci. (2023) 24. doi: 10.3390/ijms241511937. PMID: 37569313 PMC10418326

[8] Tan JK McKenzie C Mariño E Macia L Mackay CR . Metabolite-sensing G protein-coupled receptors-facilitators of diet-related immune regulation. Annu Rev Immunol. (2017) 35:371–402. doi: 10.1146/annurev-immunol-051116-052235. PMID: 28446062

[9] Daskalaki MG Tsatsanis C Kampranis SC . Histone methylation and acetylation in macrophages as a mechanism for regulation of inflammatory responses. J Cell Physiol. (2018) 233:6495–507. doi: 10.1002/jcp.26497. PMID: 29574768 PMC13482559

[10] Omdal R . The biological basis of chronic fatigue: neuroinflammation and innate immunity. Curr Opin Neurol. (2020) 33:391–6. doi: 10.1097/wco.0000000000000817. PMID: 32304437

[11] Morris G Maes M . A neuro-immune model of myalgic encephalomyelitis/chronic fatigue syndrome. Metab Brain Dis. (2013) 28:523–40. doi: 10.1007/s11011-012-9324-8. PMID: 22718491

[12] Yang W Cong Y . Gut microbiota-derived metabolites in the regulation of host immune responses and immune-related inflammatory diseases. Cell Mol Immunol. (2021) 18:866–77. doi: 10.1038/s41423-021-00661-4. PMID: 33707689 PMC8115644

[13] Komaroff AL Lipkin WI . ME/CFS and long COVID share similar symptoms and biological abnormalities: road map to the literature. Front Med Lausanne. (2023) 10:1187163. doi: 10.3389/fmed.2023.1187163. PMID: 37342500 PMC10278546

[14] Davis HE McCorkell L Vogel JM Topol EJ . Long COVID: major findings, mechanisms and recommendations. Nat Rev Microbiol. (2023) 21:133–46. doi: 10.1038/s41579-022-00846-2. PMID: 36639608 PMC9839201

[15] Strawbridge R Sartor ML Scott F Cleare AJ . Inflammatory proteins are altered in chronic fatigue syndrome-a systematic review and meta-analysis. Neurosci Biobehav Rev. (2019) 107:69–83. doi: 10.1016/j.neubiorev.2019.08.011. PMID: 31465778

[16] Recio C Lucy D Iveson P Iqbal AJ Valaris S Wynne G . The role of metabolite-sensing G protein-coupled receptors in inflammation and metabolic disease. Antioxid Redox Signal. (2018) 29:237–56. doi: 10.1089/ars.2017.7168. PMID: 29117706

[17] Sun Y Zhang Z Qiao Q Zou Y Wang L Wang T . Immunometabolic changes and potential biomarkers in CFS peripheral immune cells revealed by single-cell RNA sequencing. J Transl Med. (2024) 22:925. doi: 10.1186/s12967-024-05710-w. PMID: 39394558 PMC11468054

[18] Weyand CM Zeisbrich M Goronzy JJ . Metabolic signatures of T-cells and macrophages in rheumatoid arthritis. Curr Opin Immunol. (2017) 46:112–20. doi: 10.1016/j.coi.2017.04.010. PMID: 28538163 PMC5554742

[19] Parab A Bhatt LK . T-cell metabolism in rheumatoid arthritis: focus on mitochondrial and lysosomal dysfunction. Immunopharmacol Immunotoxicol. (2024) 46:378–84. doi: 10.1080/08923973.2024.2330645. PMID: 38478010

[20] Kim ME Lim Y Lee JS . Mitochondrial dysfunction and metabolic reprogramming in chronic inflammatory diseases: molecular insights and therapeutic opportunities. Curr Issues Mol Biol. (2025) 47. doi: 10.3390/cimb47121042. PMID: 41614806 PMC12731309

[21] Huang H Li G He Y Chen J Yan J Zhang Q . Cellular succinate metabolism and signaling in inflammation: implications for therapeutic intervention. Front Immunol. (2024) 15:1404441. doi: 10.3389/fimmu.2024.1404441. PMID: 38933270 PMC11200920

[22] Jia Y Wang L . From mechanisms to diseases: the succinate-GPR91 axis in cardiometabolic diseases. J Cardiovasc Transl Res. (2025) 18:1298–311. doi: 10.1007/s12265-025-10670-7. PMID: 40705203

[23] Mehdi S Wani SUD Krishna KL Kinattingal N Roohi TF . A review on linking stress, depression, and insulin resistance via low-grade chronic inflammation. Biochem Biophys Rep. (2023) 36:101571. doi: 10.1016/j.bbrep.2023.101571. PMID: 37965066 PMC10641573

[24] Kimble LP Khosroshahi A Brewster GS Dunbar SB Ryan D Carlson N . Associations between TCA cycle plasma metabolites and fatigue in black females with systemic lupus erythematosus: an untargeted metabolomics pilot study. Lupus. (2024) 33:948–61. doi: 10.1177/09612033241260334. PMID: 38885489 PMC11296915

[25] Qiu J Wu B Goodman SB Berry GJ Goronzy JJ Weyand CM . Metabolic control of autoimmunity and tissue inflammation in rheumatoid arthritis. Front Immunol. (2021) 12:652771. doi: 10.3389/fimmu.2021.652771. PMID: 33868292 PMC8050350

[26] Gong J Gao X Ge S Li H Wang R Zhao L . The role of cGAS-STING signalling in metabolic diseases: from signalling networks to targeted intervention. Int J Biol Sci. (2024) 20:152–74. doi: 10.7150/ijbs.84890. PMID: 38164186 PMC10750282

[27] Okinaka Y Kageyama S Goto T Sugimoto M Tomita A Aizawa Y . Metabolomic profiling of cancer-related fatigue involved in cachexia and chemotherapy. Sci Rep. (2024) 14:8329. doi: 10.1038/s41598-024-57747-y. PMID: 38594321 PMC11004174

[28] Yamano E Watanabe Y Kataoka Y . Insights into metabolite diagnostic biomarkers for myalgic encephalomyelitis/chronic fatigue syndrome. Int J Mol Sci. (2021) 22. doi: 10.3390/ijms22073423. PMID: 33810365 PMC8037376

[29] Li X Yang Y Zhang B Lin X Fu X An Y . Lactate metabolism in human health and disease. Signal Transduc Tgt Ther. (2022) 7:305. doi: 10.1038/s41392-022-01151-3. PMID: 36050306 PMC9434547

[30] Corbet C Feron O . Cancer cell metabolism and mitochondria: nutrient plasticity for TCA cycle fueling. Biochim Biophys Acta Rev Cancer. (2017) 1868:7–15. doi: 10.1016/j.bbcan.2017.01.002. PMID: 28110019

[31] Feng T Zhao X Gu P Yang W Wang C Guo Q . Adipocyte-derived lactate is a signalling metabolite that potentiates adipose macrophage inflammation via targeting PHD2. Nat Commun. (2022) 13:5208. doi: 10.1038/s41467-022-32871-3. PMID: 36064857 PMC9445001

[32] Jiang R Ren WJ Wang LY Zhang W Jiang ZH Zhu GY . Targeting lactate: an emerging strategy for macrophage regulation in chronic inflammation and cancer. Biomolecules. (2024) 14. doi: 10.3390/biom14101202. PMID: 39456135 PMC11505598

[33] Duncan EM Vita L Dibnah B Hudson BD . Metabolite-sensing GPCRs controlling interactions between adipose tissue and inflammation. Front Endocrinol Lausanne. (2023) 14:1197102. doi: 10.3389/fendo.2023.1197102. PMID: 37484963 PMC10357040

[34] Mirji G Bhat SA El Sayed M Reiser SK Gavara SP Ye Y . Aromatic microbial metabolite hippuric acid enhances inflammatory responses in macrophages via TLR-MyD88 signaling and lipid remodeling. Cell Rep. (2026) 45:116749. doi: 10.1016/j.celrep.2025.116749. PMID: 41477766 PMC12884656

[35] Zhang CS Zhang B Li M Wei X Gong K Li Z . Identification of serum metabolites enhancing inflammatory responses in COVID-19. Sci China Life Sci. (2022) 65:1971–84. doi: 10.1007/s11427-021-2099-7. PMID: 35508791 PMC9068507

[36] Hasan S Ghani N Zhao X Good J Liu CJ . Exogenous pyruvate is therapeutic against colitis by targeting cytosolic phospholipase A2. Genes Dis. (2025) 12:101571. doi: 10.1016/j.gendis.2025.101571. PMID: 40605975 PMC12221594

[37] Kurien BT Ice JA Wood RA Pharaoh G Cavett J Lewis V . Mitochondrial dysfunction and fatigue in Sjögren’s disease. RMD Open. (2025) 11. doi: 10.1136/rmdopen-2024-005046. PMID: 40274303 PMC12020762

[38] Fernandez-Guerra P Gonzalez-Ebsen AC Boonen SE Courraud J Gregersen N Mehlsen J . Bioenergetic and proteomic profiling of immune cells in myalgic encephalomyelitis/chronic fatigue syndrome patients: an exploratory study. Biomolecules. (2021) 11. doi: 10.3390/biom11070961. PMID: 34209852 PMC8301912

[39] Sabat M . Comparison of metabolic effects of mitochondrial dysfunctions in the context of vulnerability to fatigue: computer simulation study. Acta Biochim Pol. (2022) 69:513–22. doi: 10.18388/abp.2020_6432. PMID: 36049068

[40] Quintero-González DC Muñoz-Urbano M Vásquez G . Mitochondria as a key player in systemic lupus erythematosus. Autoimmunity. (2022) 55:497–505. doi: 10.1080/08916934.2022.2112181. PMID: 35978536

[41] Weichmann F Avaltroni F Burki C . Review of clinical effects and presumed mechanism of action of the French oak wood extract Robuvit. J Med Food. (2021) 24:897–907. doi: 10.1089/jmf.2020.0165. PMID: 33512270 PMC8573807

[42] Picard M McManus MJ Gray JD Nasca C Moffat C Kopinski PK . Mitochondrial functions modulate neuroendocrine, metabolic, inflammatory, and transcriptional responses to acute psychological stress. Proc Natl Acad Sci USA. (2015) 112:E6614–23. doi: 10.1073/pnas.1515733112. PMID: 26627253 PMC4672794

[43] Lee E Ozigbo AA Varon J Halma M Laezzo M Ang SP . Mitochondrial reactive oxygen species: a unifying mechanism in long COVID and spike protein-associated injury: a narrative review. Biomolecules. (2025) 15. doi: 10.3390/biom15091339. PMID: 41008646 PMC12467101

[44] Fusagawa H Sato T Yamada T Ashida Y Kimura I Naito A . Skeletal muscle endurance declines with impaired mitochondrial respiration and inadequate supply of acetyl-CoA during muscle fatigue in 5/6 nephrectomized rats. J Appl Physiol 1985. (2023) 135:731–46. doi: 10.1152/japplphysiol.00226.2023. PMID: 37560765 PMC10642514

[45] Feng LR Wolff BS Liwang J Regan JM Alshawi S Raheem S . Cancer-related fatigue during combined treatment of androgen deprivation therapy and radiotherapy is associated with mitochondrial dysfunction. Int J Mol Med. (2020) 45:485–96. doi: 10.3892/ijmm.2019.4435. PMID: 31894256 PMC6984780

[46] Kuan-Celarier A Wallander ML Hartzell J Lees B Leng XI Kramer PA . Mitochondrial bioenergetics in resilience of older adults with gynecologic cancer: design and rationale of a pilot study. GeroScience. (2026) 48(1):253–262. doi: 10.1007/s11357-025-01823-2. PMID: 40810770 PMC12972482

[47] Abe L Dantzer R . The role of FGF21 in the metabolic adjustments required for exercise capacity. Life Sci. (2025) 380:123940. doi: 10.1016/j.lfs.2025.123940. PMID: 40886940 PMC13322481

[48] Caengprasath N Gonzalez-Abuin N Shchepinova M Ma Y Inoue A Tate EW . Internalization-dependent free fatty acid receptor 2 signaling is essential for propionate-induced anorectic gut hormone release. iScience. (2020) 23:101449. doi: 10.1016/j.isci.2020.101449. PMID: 32853993 PMC7452316

[49] Kaya B Melhem H Niess JH . GPR35 in intestinal diseases: from risk gene to function. Front Immunol. (2021) 12:717392. doi: 10.3389/fimmu.2021.717392. PMID: 34790192 PMC8591220

[50] Kapolka NJ Taghon GJ Rowe JB Morgan WM Enten JF Lambert NA . DCyFIR: a high-throughput CRISPR platform for multiplexed G protein-coupled receptor profiling and ligand discovery. Proc Natl Acad Sci USA. (2020) 117:13117–26. doi: 10.1073/pnas.2000430117. PMID: 32434907 PMC7293659

[51] Lückmann M Trauelsen M Frimurer TM Schwartz TW . Structural basis for GPCR signaling by small polar versus large lipid metabolites-discovery of non-metabolite ligands. Curr Opin Cell Biol. (2020) 63:38–48. doi: 10.1016/j.ceb.2019.12.005 31951921

[52] Chen H Rosen CE González-Hernández JA Song D Potempa J Ring AM . Highly multiplexed bioactivity screening reveals human and microbiota metabolome-GPCRome interactions. Cell. (2023) 186:3095–3110.e19. doi: 10.1016/j.cell.2023.05.024. PMID: 37321219 PMC10330796

[53] Bhattarai Y Jie S Linden DR Ghatak S Mars RAT Williams BB . Bacterially derived tryptamine increases mucus release by activating a host receptor in a mouse model of inflammatory bowel disease. iScience. (2020) 23:101798. doi: 10.1016/j.isci.2020.101798. PMID: 33299969 PMC7702010

[54] Villano R Tinto F Di Marzo V . Facile and sustainable synthesis of commendamide and its analogues. Front Chem. (2022) 10:858854. doi: 10.3389/fchem.2022.858854. PMID: 35300384 PMC8921460

[55] Cho W York AG Wang R Wyche TP Piizzi G Flavell RA . N-Acyl Amides from Neisseria meningitidis and Their Role in Sphingosine Receptor Signaling. ChemBioChem. (2022) 23(22):e202200490. doi: 10.1002/cbic.202200490 36112057 PMC9762135

[56] Zhang Z Li Z . GPCR biased signaling in metabolism. Handb Exp Pharmacol. (2026) 290:319–41. doi: 10.1007/164_2025_774. PMID: 40946114

[57] Comerford I McColl SR . Atypical chemokine receptors in the immune system. Nat Rev Immunol. (2024) 24:753–69. doi: 10.1038/s41577-024-01025-5. PMID: 38714818

[58] Cao P Yang M Chang C Wu H Lu Q . Germinal center-related G protein-coupled receptors in antibody-mediated autoimmune skin diseases: from basic research to clinical trials. Clin Rev Allergy Immunol. (2022) 63:357–70. doi: 10.1007/s12016-022-08936-y. PMID: 35674978

[59] Yang X Zhang W Wang L Zhao Y Wei W . Metabolite-sensing GPCRs in rheumatoid arthritis. Trends Pharmacol Sci. (2024) 45:118–33. doi: 10.1016/j.tips.2023.12.001. PMID: 38182481

[60] Marsango S Milligan G . Regulation of the pro-inflammatory G protein-coupled receptor GPR84. Br J Pharmacol. (2024) 181:1500–8. doi: 10.22541/au.167577124.40262880/v1. PMID: 37085331

[61] Wang C Liu Y Zhang R Gong H Jiang X Xia S . Targeting the tumor immune microenvironment: GPCRs as key regulators in triple-negative breast cancer. Int Immunopharmacol. (2025) 147:113930. doi: 10.1016/j.intimp.2024.113930. PMID: 39740508

[62] Omi J Kano K Aoki J . Current knowledge on the biology of lysophosphatidylserine as an emerging bioactive lipid. Cell Biochem Biophys. (2021) 79:497–508. doi: 10.1007/s12013-021-00988-9. PMID: 34129148 PMC8551102

[63] Oguro-Igashira E Murakami M Mori R Kuwahara R Kihara T Kohara M . The pyruvate-GPR31 axis promotes transepithelial dendrite formation in human intestinal dendritic cells. Proc Natl Acad Sci USA. (2024) 121:e2318767121. doi: 10.1073/pnas.2318767121. PMID: 39432783 PMC11536072

[64] Cabral-Marques O Schimke LF Moll G Filgueiras IS Nóbile AL Adri AS . Advancing research on regulatory autoantibodies targeting GPCRs: Insights from the 5th international symposium. Autoimmun Rev. (2025) 24:103855. doi: 10.1016/j.autrev.2025.103855. PMID: 40543860

[65] Fukumoto A Suga N Nakashima M Matsuda S . GPCRs with mTOR signaling expressed in gut-brain-immune axis-cells could contribute to the treatment of neurodegenerative diseases and immune-related diseases. Discov Med. (2025) 37:608–17. doi: 10.24976/discov.med.202537195.53. PMID: 40287798

[66] Afzal MS . G proteins: binary switches in health and disease. Cent Eur J Immunol. (2020) 45:364–7. doi: 10.5114/ceji.2020.101271. PMID: 33437192 PMC7789995

[67] Sahani PA Patra R Dixit A . G protein-coupled receptors (GPCRs) in cancer prognosis and therapeutics: A comprehensive review. Int J Biol Macromol. (2025) 328:147665. doi: 10.1016/j.ijbiomac.2025.147665. PMID: 40953624

[68] Tokuyama K Yasuda H Saito M Hayashi S Kato S . Protective role of orphan G protein-coupled receptor GPR35 in the pathogenesis of colitis through regulating epithelial barrier function and immune responses. J Pharmacol Sci. (2025) 159:191–201. doi: 10.1016/j.jphs.2025.08.009. PMID: 40983462

[69] Zheng K Chen M Xu X Li P Yin C Wang J . Chemokine CXCL13-CXCR5 signaling in neuroinflammation and pathogenesis of chronic pain and neurological diseases. Cell Mol Biol Lett. (2024) 29:134. doi: 10.1186/s11658-024-00653-y. PMID: 39472796 PMC11523778

[70] Yu X Nagai J Marti-Solano M Soto JS Coppola G Babu MM . Context-specific striatal astrocyte molecular responses are phenotypically exploitable. Neuron. (2020) 108:1146–1162.e10. doi: 10.1016/j.neuron.2020.09.021. PMID: 33086039 PMC7813554

[71] Lee B Lee SM Song JW Choi JW . Gut microbiota metabolite messengers in brain function and pathology at a view of cell type-based receptor and enzyme reaction. Biomol Ther Seoul. (2024) 32:403–23. doi: 10.4062/biomolther.2024.009. PMID: 38898687 PMC11214962

[72] Muzio L Viotti A Martino G . Microglia in neuroinflammation and neurodegeneration: From understanding to therapy. Front Neurosci. (2021) 15:742065. doi: 10.3389/fnins.2021.742065. PMID: 34630027 PMC8497816

[73] Ajoolabady A Kim B Abdulkhaliq AA Ren J Bahijri S Tuomilehto J . Dual role of microglia in neuroinflammation and neurodegenerative diseases. Neurobiol Dis. (2025) 216:107133. doi: 10.1016/j.nbd.2025.107133. PMID: 41052547

[74] Maziz MNH Chakravarthi S Aung T Htoo PM Shwe WH Gupalo S . Microglia-mediated neuroinflammation through phosphatidylinositol 3-kinase signaling causes cognitive dysfunction. Int J Mol Sci. (2025) 26. doi: 10.3390/ijms26157212. PMID: 40806341 PMC12346505

[75] Denver P Cunningham C . Microglial activation and neuroinflammation in acute and chronic cognitive deficits in sepsis. Neuropharmacology. (2025) 267:110285. doi: 10.1016/j.neuropharm.2024.110285. PMID: 39746541

[76] Lee JS Sato W Son CG . Brain-regional characteristics and neuroinflammation in ME/CFS patients from neuroimaging: A systematic review and meta-analysis. Autoimmun Rev. (2024) 23:103484. doi: 10.1016/j.autrev.2023.103484. PMID: 38016575

[77] Fan J Jiao J Chang HQ Zhong DL Liu XB Li J . Myalgic encephalomyelitis/chronic fatigue syndrome (ME/CFS): diagnosis and management. J Transl Med. (2025) 24:62. doi: 10.1186/s12967-025-07506-y. PMID: 41366804 PMC12801797

[78] Dudova D Bozhkova M Petrov S Nikolova R Kalfova T Ivanovska M . Insights into the complex biological network underlying myalgic encephalomyelitis/chronic fatigue syndrome. Int J Mol Sci. (2025) 27. doi: 10.3390/ijms27010268. PMID: 41516145 PMC12785471

[79] Lee HG Lee JH Flausino LE Quintana FJ . Neuroinflammation: An astrocyte perspective. Sci Transl Med. (2023) 15:eadi7828. doi: 10.1126/scitranslmed.adi7828. PMID: 37939162

[80] Zhao Y Huang Y Cao Y Yang J . Astrocyte-mediated neuroinflammation in neurological conditions. Biomolecules. (2024) 14. doi: 10.3390/biom14101204. PMID: 39456137 PMC11505625

[81] Singhal G Singhal S Baune BT . Role of astrocyte in neuroinflammation-induced loss in neuroplasticity and subsequent onset of depression: A systematic review. Neuroprotection. (2025) 3:206–25. doi: 10.1002/nep3.70009. PMID: 41394305 PMC12699557

[82] Babin KM Gostynska SE Karim JA Pioszak AA . Variable CGRP family peptide signaling durations and the structural determinants thereof. Biochem Pharmacol. (2024) 224:116235. doi: 10.1016/j.bcp.2024.116235. PMID: 38670438 PMC11102832

[83] Babin KM Kilinc C Gostynska SE Dickson A Pioszak AA . Characterization of the two-domain peptide binding mechanism of the human CGRP receptor for CGRP and the ultrahigh affinity ssCGRP variant. Biochemistry. (2025) 64:1770–87. doi: 10.1021/acs.biochem.4c00812. PMID: 40172014 PMC12004451

[84] Kasatkina LA Rittchen S Sturm EM . Neuroprotective and immunomodulatory action of the endocannabinoid system under neuroinflammation. Int J Mol Sci. (2021) 22. doi: 10.3390/ijms22115431. PMID: 34063947 PMC8196612

[85] Esch T Kream RM Stefano GB . Emerging regulatory roles of opioid peptides, endogenous morphine, and opioid receptor subtypes in immunomodulatory processes: Metabolic, behavioral, and evolutionary perspectives. Immunol Lett. (2020) 227:28–33. doi: 10.1016/j.imlet.2020.08.007. PMID: 32827633

[86] Li S Li N He J Zhou R Lu Z Tao YJ . Molecular basis of KAT2A selecting acyl-CoA cofactors for histone modifications. Res Wash D C. (2023) 6:109. doi: 10.34133/research.0109. PMID: 37040526 PMC10076270

[87] Xiao Y Li W Yang H Pan L Zhang L Lu L . HBO1 is a versatile histone acyltransferase critical for promoter histone acylations. Nucleic Acids Res. (2021) 49:8037–59. doi: 10.1093/nar/gkab607. PMID: 34259319 PMC8661427

[88] Trefely S Lovell CD Snyder NW Wellen KE . Compartmentalised acyl-CoA metabolism and roles in chromatin regulation. Mol Metab. (2020) 38:100941. doi: 10.1016/j.molmet.2020.01.005. PMID: 32199817 PMC7300382

[89] Nitsch S Zorro Shahidian L Schneider R . Histone acylations and chromatin dynamics: concepts, challenges, and links to metabolism. EMBO Rep. (2021) 22:e52774. doi: 10.15252/embr.202152774. PMID: 34159701 PMC8406397

[90] Trefely S Huber K Liu J Noji M Stransky S Singh J . Quantitative subcellular acyl-CoA analysis reveals distinct nuclear metabolism and isoleucine-dependent histone propionylation. Mol Cell. (2022) 82:447–462.e6. doi: 10.1016/j.molcel.2021.11.006. PMID: 34856123 PMC8950487

[91] Jo C Park S Oh S Choi J Kim EK Youn HD . Histone acylation marks respond to metabolic perturbations and enable cellular adaptation. Exp Mol Med. (2020) 52:2005–19. doi: 10.1038/s12276-020-00539-x. PMID: 33311704 PMC8080766

[92] Varner EL Trefely S Bartee D von Krusenstiern E Izzo L Bekeova C . Quantification of lactoyl-CoA (lactyl-CoA) by liquid chromatography mass spectrometry in mammalian cells and tissues. Open Biol. (2020) 10:200187. doi: 10.1098/rsob.200187. PMID: 32961073 PMC7536085

[93] Fu Y Yu J Li F Ge S . Oncometabolites drive tumorigenesis by enhancing protein acylation: from chromosomal remodelling to nonhistone modification. J Exp Clin Cancer Res. (2022) 41:144. doi: 10.1186/s13046-022-02338-w. PMID: 35428309 PMC9013066

[94] Li X Yu T Li X He X Zhang B Yang Y . Role of novel protein acylation modifications in immunity and its related diseases. Immunology. (2024) 173:53–75. doi: 10.1111/imm.13822. PMID: 38866391

[95] Trujillo MN Jennings EQ Hoffman EA Zhang H Phoebe AM Mastin GE . Lactoylglutathione promotes inflammatory signaling in macrophages through histone lactoylation. Mol Metab. (2024) 81:101888. doi: 10.1016/j.molmet.2024.101888. PMID: 38307385 PMC10869261

[96] Shang S Liu J Hua F . Protein acylation: mechanisms, biological functions and therapeutic targets. Signal Transduc Tgt Ther. (2022) 7:396. doi: 10.1038/s41392-022-01245-y. PMID: 36577755 PMC9797573

[97] Mutlu B Puigserver P . GCN5 acetyltransferase in cellular energetic and metabolic processes. Biochim Biophys Acta Gene Regul Mech. (2021) 1864:194626. doi: 10.1016/j.bbagrm.2020.194626. PMID: 32827753 PMC7854474

[98] Certo M Elkafrawy H Pucino V Cucchi D Cheung KCP Mauro C . Endothelial cell and T-cell crosstalk: Targeting metabolism as a therapeutic approach in chronic inflammation. Br J Pharmacol. (2021) 178:2041–59. doi: 10.1111/bph.15002. PMID: 31999357 PMC8246814

[99] Wang W Hou S Han M Zheng Y Ma R Liu Z . Research progress in metabolic reprogramming of endothelial cells and T cells and potential therapeutic targets for chronic inflammation. Xi Bao Yu Fen Zi Mian Yi Xue Za Zhi. (2021) 37(11):1038–1044. 34809744

[100] Garcia-Segura ME Pluchino S Peruzzotti-Jametti L . Metabolic control of microglia. Adv Neurobiol. (2024) 37:607–22. doi: 10.1007/978-3-031-55529-9_34. PMID: 39207716

[101] Miallot R Millet V Galland F Naquet P . The vitamin B5/coenzyme A axis: A target for immunomodulation? Eur J Immunol. (2023) 53:e2350435. doi: 10.1002/eji.202350435. PMID: 37482959

[102] Patiño-Martinez E Kaplan MJ . Immunometabolism in systemic lupus erythematosus. Nat Rev Rheumatol. (2025) 21:377–95. doi: 10.1038/s41584-025-01267-0. PMID: 40524030

[103] Rodriguez-Coira J Villaseñor A Izquierdo E Huang M Barker-Tejeda TC Radzikowska U . The importance of metabolism for immune homeostasis in allergic diseases. Front Immunol. (2021) 12:692004. doi: 10.3389/fimmu.2021.692004. PMID: 34394086 PMC8355700

[104] Straub RH Pongratz G Buttgereit F Gaber T . Energy metabolism of the immune system: Consequences in chronic inflammation. Z Rheumatol. (2023) 82:479–90. doi: 10.1007/978-3-662-68904-2_4. PMID: 37488246

[105] Ito A Suganami T . Lipid metabolism in myeloid cell function and chronic inflammatory diseases. Front Immunol. (2024) 15:1495853. doi: 10.3389/fimmu.2024.1495853. PMID: 39911578 PMC11794072

[106] Ohashi T Inoue N Aoki M . The Warburg effect and M2 macrophage polarization in head and neck cancer. Gan To Kagaku Ryoho. (2020) 47(1):6–10. 32381853

[107] Xiong Y Zhou J Wang J Huang H . How lactate and lactylation shape the immunity system in atherosclerosis (review). Int J Mol Med. (2025) 56. doi: 10.3892/ijmm.2025.5604. PMID: 40747658 PMC12339161

[108] Liu X Xiang R Fang X Wang G Zhou Y . Advances in metabolic regulation of macrophage polarization state. Immunol Invest. (2024) 53:416–36. doi: 10.1080/08820139.2024.2302828. PMID: 38206296

[109] Liu S Yang J Wu Z . The regulatory role of α-ketoglutarate metabolism in macrophages. Mediators Inflammation. (2021) 2021:5577577. doi: 10.1155/2021/5577577. PMID: 33859536 PMC8024083

[110] Lun H Li P Li J Liu F . The effect of intestinal flora metabolites on macrophage polarization. Heliyon. (2024) 10:e35755. doi: 10.1016/j.heliyon.2024.e35755. PMID: 39170251 PMC11337042

[111] Yang Y Karampoor S Mirzaei R Borozdkin L Zhu P . The interplay between microbial metabolites and macrophages in cardiovascular diseases: A comprehensive review. Int Immunopharmacol. (2023) 121:110546. doi: 10.1016/j.intimp.2023.110546. PMID: 37364331

[112] Li L Tian Y . The role of metabolic reprogramming of tumor-associated macrophages in shaping the immunosuppressive tumor microenvironment. BioMed Pharmacother. (2023) 161:114504. doi: 10.1016/j.biopha.2023.114504. PMID: 37002579

[113] Garavaglia B Vallino L Ferraresi A Amoruso A Pane M Isidoro C . Probiotic-derived metabolites from Lactiplantibacillus plantarum OC01 reprogram tumor-associated macrophages to an inflammatory anti-tumoral phenotype: Impact on colorectal cancer cell proliferation and migration. Biomedicines. (2025) 13. doi: 10.3390/biomedicines13020339. PMID: 40002754 PMC11853712

[114] Luo Y Xu H Xiong S Ke J . Understanding myalgic encephalomyelitis/chronic fatigue syndrome physical fatigue through the perspective of immunosenescence. Compr Physiol. (2025) 15:e70056. doi: 10.1002/cph4.70056. PMID: 41017304

[115] Fenimore JM Springer DA Romero ME Edmondson EF McVicar DW Yanpallewar S . IFN-γ and androgens disrupt mitochondrial function in murine myocytes. J Pathol. (2023) 260:276–88. doi: 10.1002/path.6081. PMID: 37185821 PMC10330777

[116] Xiao C Peng G Conneely KN Zhao H Felger JC Wommack EC . DNA methylation profiles of cancer-related fatigue associated with markers of inflammation and immunometabolism. Mol Psychiatry. (2025) 30:76–83. doi: 10.1038/s41380-024-02652-z. PMID: 38977918

[117] Gupta G Buonsenso D Wood J Mohandas S Warburton D . Mechanistic insights into long covid: Viral persistence, immune dysregulation, and multi-organ dysfunction. Compr Physiol. (2025) 15:e70019. doi: 10.1002/cph4.70019. PMID: 40474772

[118] Molfino A Imbimbo G Gallicchio C Muscaritoli M . Tryptophan metabolism and kynurenine metabolites in cancer: Systemic nutritional and metabolic implications. Curr Opin Clin Nutr Metab Care. (2024) 27:316–21. doi: 10.1097/mco.0000000000001021. PMID: 38386476

[119] Fosså A Smeland KH Fluge Ø Tronstad KJ Loge JH Midttun Ø . Metabolic analysis of amino acids and vitamin B6 pathways in lymphoma survivors with cancer related chronic fatigue. PLoS One. (2020) 15(1):e0227384. doi: 10.1371/journal.pone.0227384 31923274 PMC6953873

[120] Nkiliza A Parks M Cseresznye A Oberlin S Evans JE Darcey T . Sex-specific plasma lipid profiles of ME/CFS patients and their association with pain, fatigue, and cognitive symptoms. J Transl Med. (2021) 19:370. doi: 10.1186/s12967-021-03035-6. PMID: 34454515 PMC8401202

[121] Vollbracht C Kraft K . Oxidative stress and hyper-inflammation as major drivers of severe COVID-19 and long COVID: Implications for the benefit of high-dose intravenous vitamin C. Front Pharmacol. (2022) 13:899198. doi: 10.3389/fphar.2022.899198. PMID: 35571085 PMC9100929

[122] Ma S Ono M Mizugaki A Kato H Miyashita M Suzuki K . Cystine/glutamine mixture supplementation attenuated fatigue during endurance exercise in healthy young men by enhancing fatty acid utilization. Sports Bsl. (2022) 10. doi: 10.3390/sports10100147. PMID: 36287760 PMC9610368

[123] Liu Y Hu Y Ma B Wang Z Wei B . Gut microbiota and exercise: Probiotics to modify the composition and roles of the gut microbiota in the context of 3P medicine. Microb Ecol. (2025) 88:38. doi: 10.1007/s00248-025-02529-w. PMID: 40319213 PMC12049406

[124] Yoo L Mendoza D Richard AJ Stephens JM . KAT8 beyond acetylation: A survey of its epigenetic regulation, genetic variability, and implications for human health. Genes Bsl. (2024) 15. doi: 10.3390/genes15050639. PMID: 38790268 PMC11121512

[125] Li X Huang L Pan L Wang B Pan L . CRISPR/dCas9-mediated epigenetic modification reveals differential regulation of histone acetylation on Aspergillus Niger secondary metabolite. Microbiol Res. (2021) 245:126694. doi: 10.1016/j.micres.2020.126694. PMID: 33482403

[126] Egger AS Rauch E Sharma S Kipura T Hotze M Mair T . Linking metabolism and histone acetylation dynamics by integrated metabolic flux analysis of acetyl-CoA and histone acetylation sites. Mol Metab. (2024) 90:102032. doi: 10.1016/j.molmet.2024.102032. PMID: 39305948 PMC11492620

[127] Russo M Pileri F Ghisletti S . Novel insights into the role of acetyl-CoA producing enzymes in epigenetic regulation. Front Endocrinol Lausanne. (2023) 14:1272646. doi: 10.3389/fendo.2023.1272646. PMID: 37842307 PMC10570720

[128] Beentjes SV Miralles Méharon A Kaczmarczyk J Cassar A Samms GL Hejazi NS . Replicated blood-based biomarkers for myalgic encephalomyelitis not explicable by inactivity. EMBO Mol Med. (2025) 17:1868–91. doi: 10.1038/s44321-025-00258-8. PMID: 40537675 PMC12254397

[129] Xiao C Fedirko V Claussen H Richard Johnston H Peng G Paul S . Circulating short chain fatty acids and fatigue in patients with head and neck cancer: A longitudinal prospective study. Brain Behav Immun. (2023) 113:432–43. doi: 10.1016/j.bbi.2023.07.025. PMID: 37543249 PMC10528227

[130] Xiong R Aiken E Caldwell R Vernon SD Kozhaya L Gunter C . AI-driven multi-omics modeling of myalgic encephalomyelitis/chronic fatigue syndrome. Nat Med. (2025) 31:2991–3001. doi: 10.1038/s41591-025-03788-3. PMID: 40715814 PMC12416096

[131] Meihua S Jiahui J Yujia L Shuang Z Jingjing Z . Research on sweat metabolomics of athlete’s fatigue induced by high intensity interval training. Front Physiol. (2023) 14:1269885. doi: 10.3389/fphys.2023.1269885. PMID: 38033334 PMC10684900

[132] Scholey E Apps MAJ . Fatigue: Tough days at work change your prefrontal metabolites. Curr Biol. (2022) 32:R876–9. doi: 10.1016/j.cub.2022.06.088. PMID: 35998595

[133] Guo C Che X Briese T Ranjan A Allicock O Yates RA . Deficient butyrate-producing capacity in the gut microbiome is associated with bacterial network disturbances and fatigue symptoms in ME/CFS. Cell Host Microbe. (2023) 31:288–304.e8. doi: 10.1016/j.chom.2023.01.004. PMID: 36758522 PMC10183837

[134] Hu M Zheng K Zhang L Kan Y Zhao J Chen D . Therapeutic strategies targeting aerobic glycolysis in cancer and dynamic monitoring of associated metabolites. Cells. (2025) 14. doi: 10.3390/cells14161288. PMID: 40862767 PMC12385018

[135] Won SM Park E Jeong JJ Ganesan R Gupta H Gebru YA . The gut microbiota-derived immune response in chronic liver disease. Int J Mol Sci. (2021) 22. doi: 10.3390/ijms22158309. PMID: 34361075 PMC8347749

[136] Yu Y Jin F Wang L Cheng J Pan S . Role of gut microbiota and metabolite remodeling on the development and management of rheumatoid arthritis: A narrative review. Vet Sci. (2025) 12. doi: 10.3390/vetsci12070642. PMID: 40711302 PMC12298641

[137] Cosín-Roger J Ortiz-Masia D BarraChina MD Calatayud S . Metabolite sensing GPCRs: Promising therapeutic targets for cancer treatment? Cells. (2020) 9. doi: 10.3390/cells9112345. PMID: 33113952 PMC7690732

[138] Fu Q Cat A Zheng YG . New histone lysine acylation biomarkers and their roles in epigenetic regulation. Curr Protoc. (2023) 3:e746. doi: 10.1002/cpz1.746. PMID: 37098732 PMC13528283

[139] Gao Y Siyu Z Zhang X Du Y Ni T Hao S . Crosstalk between metabolic and epigenetic modifications during cell carcinogenesis. iScience. (2024) 27:111359. doi: 10.1016/j.isci.2024.111359. PMID: 39660050 PMC11629229

[140] Guo L Du Y Li H He T Yao L Yang G . Metabolites-mediated posttranslational modifications in cardiac metabolic remodeling: Implications for disease pathology and therapeutic potential. Metabolism. (2025) 165:156144. doi: 10.1016/j.metabol.2025.156144. PMID: 39864796

[141] Bhinderwala F Roth HE Filipi M Jack S Powers R . Potential metabolite biomarkers of multiple sclerosis from multiple biofluids. ACS Chem Neurosci. (2024) 15:1110–24. doi: 10.1021/acschemneuro.3c00678. PMID: 38420772 PMC11586083

[142] Lorente JS Sokolov AV Ferguson G Schiöth HB Hauser AS Gloriam DE . GPCR drug discovery: New agents, targets and indications. Nat Rev Drug Discov. (2025) 24:458–79. doi: 10.1038/s41573-025-01139-y. PMID: 40033110

[143] Singh A Shadangi S Rana S . G-protein coupled receptors in neuroinflammation, neuropharmacology, and therapeutics. Biochem Pharmacol. (2025) 242:117301. doi: 10.1016/j.bcp.2025.117301. PMID: 40912369

[144] Duan J He XH Li SJ Xu HE . Cryo-electron microscopy for GPCR research and drug discovery in endocrinology and metabolism. Nat Rev Endocrinol. (2024) 20:349–65. doi: 10.1038/s41574-024-00957-1. PMID: 38424377

[145] Ji RL Wang Z Zhao JJ . Beyond G protein and arrestin: GRK2-biased β_2_AR signaling. Trends Pharmacol Sci. (2025) 46:928–30. doi: 10.1016/j.tips.2025.07.010. PMID: 40752997 PMC12321243

[146] Oliveira de Souza C Sun X Oh D . Metabolic functions of G protein-coupled receptors and β-arrestin-mediated signaling pathways in the pathophysiology of type 2 diabetes and obesity. Front Endocrinol Lausanne. (2021) 12:715877. doi: 10.3389/fendo.2021.715877. PMID: 34497585 PMC8419444

[147] Cho YY Kim S Kim P Jo MJ Park SE Choi Y . G-protein-coupled receptor (GPCR) signaling and pharmacology in metabolism: Physiology, mechanisms, and therapeutic potential. Biomolecules. (2025) 15. doi: 10.3390/biom15020291. PMID: 40001594 PMC11852853

[148] Lin Y Wang Y . Orphan GPCRs regulate the metabolic liver diseases through mediating the crosstalk between the liver and immune cells: Mechanisms and therapeutics. Eur J Med Chem. (2025) 297:117906. doi: 10.1016/j.ejmech.2025.117906. PMID: 40592182

[149] Kugawa M Kawakami K Kise R Suomivuori CM Tsujimura M Kobayashi K . Structural insights into lipid chain-length selectivity and allosteric regulation of FFA2. Nat Commun. (2025) 16:2809. doi: 10.1038/s41467-025-57983-4. PMID: 40140663 PMC11947310

[150] Clausen Lind A De Castro Gomes D Bisquert R Mårtensson J Sundqvist M Forsman H . Development of a yeast-based sensor platform for evaluation of ligands recognized by the human free fatty acid 2 receptor. FEMS Yeast Res. (2025) 25. doi: 10.1093/femsyr/foaf001. PMID: 39824656 PMC11781196

[151] Davenport AP Scully CCG de Graaf C Brown AJH Maguire JJ . Advances in therapeutic peptides targeting G protein-coupled receptors. Nat Rev Drug Discov. (2020) 19:389–413. doi: 10.1038/s41573-020-0062-z. PMID: 32494050

[152] Downey ML Peralta-Yahya P . Technologies for the discovery of G protein-coupled receptor-targeting biologics. Curr Opin Biotechnol. (2024) 87:103138. doi: 10.1016/j.copbio.2024.103138. PMID: 38728825 PMC11250939

[153] Freeberg KA Udovich CC Martens CR Seals DR Craighead DH . Dietary supplementation with NAD+-boosting compounds in humans: Current knowledge and future directions. J Gerontol A Biol Sci Med Sci. (2023) 78:2435–48. doi: 10.1093/gerona/glad106. PMID: 37068054 PMC10692436

[154] Gindri IM Ferrari G Pinto LPS Bicca J Dos Santos IK Dallacosta D . Evaluation of safety and effectiveness of NAD in different clinical conditions: a systematic review. Am J Physiol Endocrinol Metab. (2024) 326:E417–e27. doi: 10.1152/ajpendo.00242.2023. PMID: 37971292

[155] Kimura T Singh S Tanaka N Umemura T . Role of G protein-coupled receptors in hepatic stellate cells and approaches to anti-fibrotic treatment of non-alcoholic fatty liver disease. Front Endocrinol Lausanne. (2021) 12:773432. doi: 10.3389/fendo.2021.773432. PMID: 34938271 PMC8685252

[156] Sun X Brueck L Yang D Sheets PL Zhou B Ren H . Leptin and G-protein coupled receptor (GPCR) signaling: Therapeutic potential in obesity. J Biol Chem. (2025) 301:110768. doi: 10.1016/j.jbc.2025.110768. PMID: 41016636 PMC12596694

[157] Zhu H Conley JM Karavadhi S LaVigne JE Watts VJ Sun H . Discovery of novel and selective GPR17 antagonists as pharmacological tools for developing new therapeutic strategies in diabetes and obesity. Eur J Med Chem. (2025) 295:117794. doi: 10.1016/j.ejmech.2025.117794. PMID: 40460721 PMC12459614

[158] Kumari P Dvorácskó S Enos MD Ramesh K Lim D Hassan SA . Structural mechanism of CB(1)R binding to peripheral and biased inverse agonists. Nat Commun. (2024) 15:10694. doi: 10.2210/pdb9b9y/pdb. PMID: 39695122 PMC11655885

[159] Schreiber AR Kagihara JA Corr BR Davis SL Lieu C Kim SS . First-in-human dose-escalation study of the novel oral depsipeptide class I-targeting HDAC inhibitor Bocodepsin (OKI-179) in patients with advanced solid tumors. Cancers Bsl. (2023) 16. doi: 10.3390/cancers16010091. PMID: 38201519 PMC10778198

[160] Sun Y Li D Su Y Zhao H Pang W Zhao W . Protective effect of hydrogen sulfide is mediated by negative regulation of epigenetic histone acetylation in Parkinson’s disease. Arch Med Sci. (2023) 19:1124–35. doi: 10.5114/aoms.2020.93121. PMID: 37560727 PMC10408026

[161] Dominguez M Brüne B Namgaladze D . Exploring the role of ATP-citrate lyase in the immune system. Front Immunol. (2021) 12:632526. doi: 10.3389/fimmu.2021.632526. PMID: 33679780 PMC7930476

[162] Nguyen T Sridaran D Chouhan S Weimholt C Wilson A Luo J . Histone H2A Lys130 acetylation epigenetically regulates androgen production in prostate cancer. Nat Commun. (2023) 14:3357. doi: 10.1038/s41467-023-38887-7. PMID: 37296155 PMC10256812

[163] Evans LW Stratton MS Ferguson BS . Dietary natural products as epigenetic modifiers in aging-associated inflammation and disease. Nat Prod Rep. (2020) 37:653–76. doi: 10.1039/c9np00057g. PMID: 31993614 PMC7577396

[164] Zhou K Liu M Wang Y Liu H Manor B Bao D . Effects of molecular hydrogen supplementation on fatigue and aerobic capacity in healthy adults: A systematic review and meta-analysis. Front Nutr. (2023) 10:1094767. doi: 10.3389/fnut.2023.1094767. PMID: 36819697 PMC9934906

[165] Tan Y Xie Y Dong G Yin M Shang Z Zhou K . The effect of 14-day consumption of hydrogen-rich water alleviates fatigue but does not ameliorate dyspnea in long-COVID patients: A pilot, single-blind, and randomized, controlled trial. Nutrients. (2024) 16. doi: 10.3390/nu16101529. PMID: 38794767 PMC11123997

[166] Zhang H Zhao C Hou J Su P Yang Y Xia B . Red ginseng extract improves skeletal muscle energy metabolism and mitochondrial function in chronic fatigue mice. Front Pharmacol. (2022) 13:1077249. doi: 10.3389/fphar.2022.1077249. PMID: 36618917 PMC9816794

[167] Dooley M . Biomarkers over time: From visual contrast sensitivity to transcriptomics in differentiating chronic inflammatory response syndrome and myalgic encephalomyelitis/chronic fatigue syndrome. Int J Mol Sci. (2025) 26. doi: 10.20944/preprints202506.1142.v1 PMC12346794 40806417

[168] Hunter E Alshaker H Bundock O Weston C Bautista S Gebregzabhar A . Development and validation of blood-based diagnostic biomarkers for Myalgic Encephalomyelitis/Chronic Fatigue Syndrome (ME/CFS) using EpiSwitch(®) 3-dimensional genomic regulatory immuno-genetic profiling. J Transl Med. (2025) 23:1048. doi: 10.1186/s12967-025-07203-w. PMID: 41057909 PMC12506310

[169] El-Sehrawy A Ayoub II Uthirapathy S Ballal S Gabble BC Singh A . The microbiota-gut-brain axis in myalgic encephalomyelitis/chronic fatigue syndrome: a narrative review of an emerging field. Eur J Transl Myol. (2025) 35. doi: 10.4081/ejtm.2025.13690. PMID: 39937103 PMC12038572

